# Regulators of male and female sexual development are critical for the transmission of a malaria parasite

**DOI:** 10.1016/j.chom.2022.12.011

**Published:** 2023-01-11

**Authors:** Andrew J.C. Russell, Theo Sanderson, Ellen Bushell, Arthur M. Talman, Burcu Anar, Gareth Girling, Mirjam Hunziker, Robyn S. Kent, Julie S. Martin, Tom Metcalf, Ruddy Montandon, Vikash Pandey, Mercedes Pardo, A. Brett Roberts, Claire Sayers, Frank Schwach, Jyoti S. Choudhary, Julian C. Rayner, Thierry Voet, Katarzyna K. Modrzynska, Andrew P. Waters, Mara K.N. Lawniczak, Oliver Billker

**Affiliations:** 1 Wellcome Sanger Institute, Hinxton CB10 1SA, UK; 2 Francis Crick Institute, 1 Midland Road, London NW1 1AT, UK; 3 Laboratory for Molecular Infection Medicine Sweden, Umeå University, Umeå 90187, Sweden; 4 Department of Molecular Biology, Umeå University, Umeå 90187, Sweden; 5 MIVEGEC, University of Montpellier, IRD, CNRS, Montpellier, France; 6 Wellcome Centre for Integrative Parasitology, Institute of Infection, Immunity, and Inflammation, University of Glasgow, Glasgow G12 8TA, UK; 7 The Institute of Cancer Research, London SW3 6JB, UK; 8 Cambridge Institute for Medical Research, University of Cambridge, Cambridge CB2 0XY, UK; 9 Department of Human Genetics, University of Leuven, KU Leuven, B-3000 Leuven, Belgium; 10 KU Leuven Institute for Single Cell Omics, LISCO, KU Leuven, 3000 Leuven, Belgium; 11 Department of Microbiology and Molecular Genetics, University of Vermont Larner College of Medicine, Burlington, VT 05405, USA

## Abstract

Malaria transmission to mosquitoes requires a developmental switch in asexually dividing blood-stage parasites to sexual reproduction. In *Plasmodium berghei*, the transcription factor AP2-G is required and sufficient for this switch, but how a particular sex is determined in a haploid parasite remains unknown. Using a global screen of barcoded mutants, we here identify genes essential for the formation of either male or female sexual forms and validate their importance for transmission. High-resolution single-cell transcriptomics of ten mutant parasites portrays the developmental bifurcation and reveals a regulatory cascade of putative gene functions in the determination and subsequent differentiation of each sex. A male-determining gene with a LOTUS/OST-HTH domain as well as the protein interactors of a female-determining zinc-finger protein indicate that germ-granule-like ribonucleoprotein complexes complement transcriptional processes in the regulation of both male and female development of a malaria parasite.

## Graphical abstract

**Graphical abstract:**
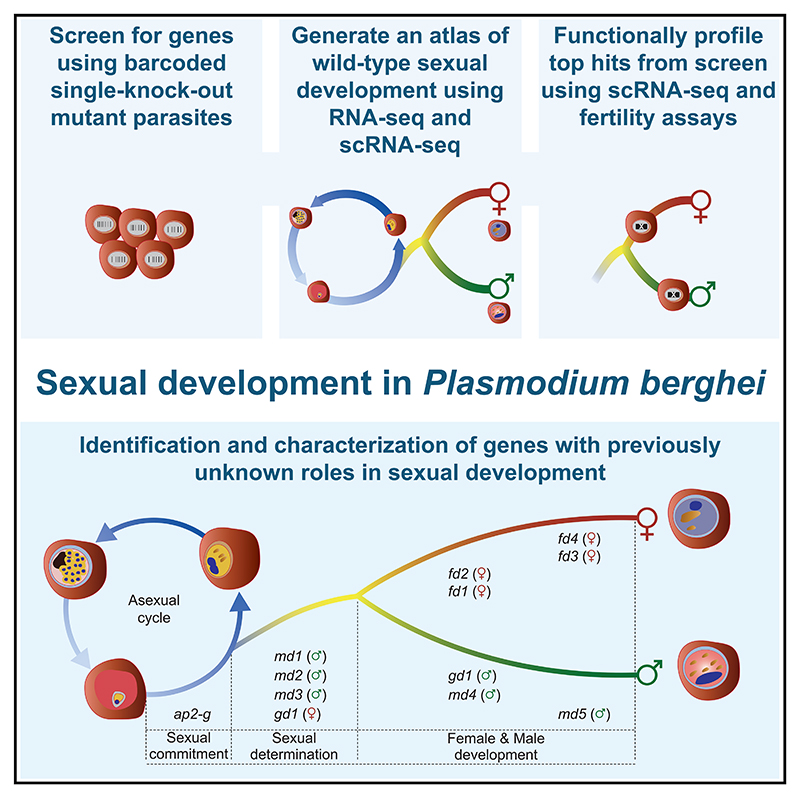

## Introduction

The transmission of malaria parasites to their mosquito vectors requires that a subset of blood-stage parasites switch from repeated asexual replication to sexual development. Epigenetically controlled expression of the transcription factor AP2-G is essential for asexual parasites to commit to sexual development,^1–4^ but the events that regulate the subsequent differentiation of sexually committed parasites into either sex are not understood. Although induced overexpression of AP2-G is sufficient to reprogram asexual parasites for sexual development experimentally^5,6^ it remains unknown how this single transcription factor generates two diverging gene expression programs that rapidly lead to male and female gametocytes.

Gametocyte sex ratios differ both between infected hosts and during the course of individual infections in ways that affect transmission to the vector and thereby the epidemiology of malaria.^7–10^ Gametocyte sex determination cannot involve sex chromosomes or inherited mating type loci because asexual *Plasmodium* blood stages are haploid and because competent clones retain the ability to produce both male and female gametocytes. Together, these observations suggest an epigenetic (i.e., non-chromosomal) mechanism for the emergence and differentiation of different sexes that is responsive to environmental regulation. Although sex is thought to have evolved only once in the ancestral eukaryote,^11^ mechanisms for how different sexes are determined evolve rapidly.^12^ As a result, none of the genes involved in equivalent determinations in other eukaryotes^12^ have clear homologs in the Apicomplexa, the phylum of divergent eukaryotes to which malaria parasites belong. In light of the absence of clear candidates based on sequence homology, a genome-wide screen is ideally suited to identify the genes involved in sex determination in *Plasmodium*.

Downstream of sex determination, another AP2 domain transcription factor, AP2-FG binds to the promoters of many female-specific genes and is required for the establishment of the full female gene expression profile.^13^ Other late events in gametocyte differentiation and post-fertilization development require the regulation of RNA metabolism and translation. Examples include the CCR4/Not complex, which in *Plasmodium* can be assembled on one of two scaffold proteins, one of which has gametocyte-specific functions in *P. yoelii*^14^ and the DDX6 family RNA helicase DOZI (development of zygote inhibited), which in *P. berghei* female gametocytes is critical for the preservation and translational repression of transcripts with post-fertilization functions.^15^ Although all these mechanisms are required to produce fertile gametocytes, none have been found to be involved in regulating sex ratio or establishing a particular sex.

In the rodent parasite *P. berghei*, pools of barcoded mutants can now be screened to discover gene functions in an unbiased manner,^16–18^ and phenotypes observed in asexual blood-stage *P. berghei* have been largely predictive of those in *P. falciparum*.^16,19^ Here, we have used a bar-seq screen with a reporter line to screen for genes required for the formation of male and female gametocytes. We subsequently performed a bulk RNA sequencing (RNA-seq) time course of gene expression following AP2-G induction to further refine our selection of candidate genes. Next, we used single-cell transcriptomics to map the wild-type differentiation of these lineages at high temporal resolution, and finally, profiled parasites with single knockout mutations of ten candidate genes by single-cell RNA sequencing (scRNA-seq) to place these screen hits in context. We find that parasites express markers indicative of their eventual sex early in the developmental bifurcation, and by disrupting these genes and characterizing mutants, we identify essential components of the male and female transcriptional programs.

## Results

### A systematic screen identifies sexual development genes

**Figure 1:**
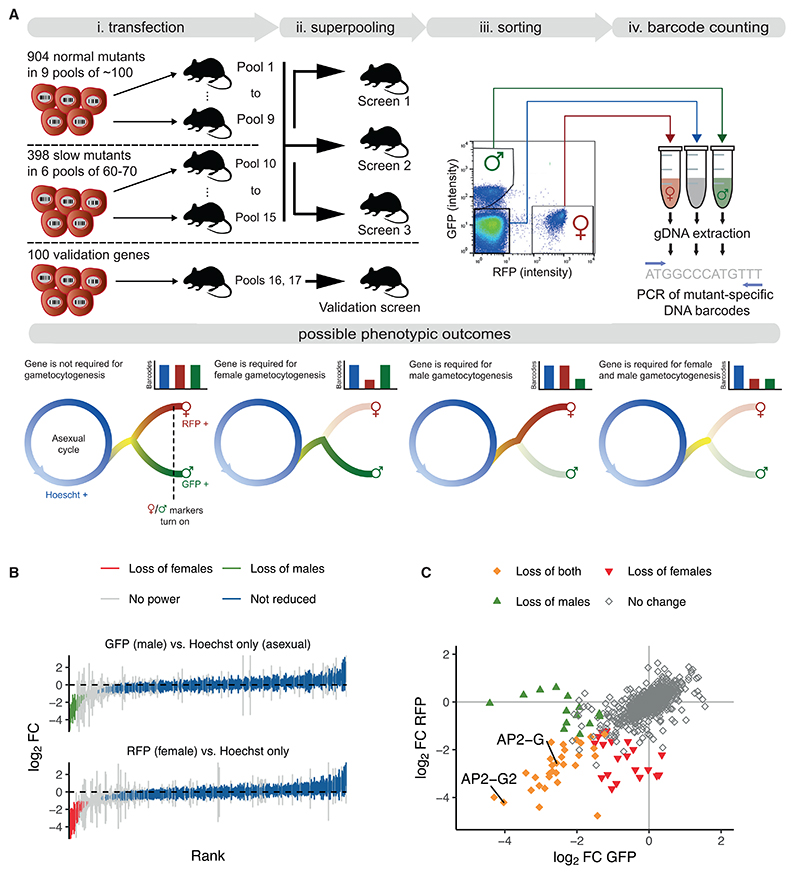
A bar-seq screen for sexual reporter expression in *P. berghei* (A) Schematic overview showing how *Plasmo*GEM vectors were allocated to pools for transfection and the resulting mutants combined into superpools for sorting on reporter expression. Duplicate barcode PCRs were performed for each sorted population and converted into sequencing libraries for barcode counting. Possible phenotypic outcomes are illustrated, using sexual marker expression as a proxy for gametocytogenesis genes that affect sexual development after the sex-specific promoters are turned on (dashed line) are unlikely to be identified in the screen. (B) All robustly quantified mutants are ranked by the degree to which cells expressing either the male or the female reporter gene were underrepresented. Error bars show standard deviations from at least 4 independent screening experiments. (C) Combined results from both reporters, showing filled symbols where the underrepresentation was significant for either one or both sexes. Two highlighted mutants in ApiAP2 genes confirm the expected loss of both markers from the population, as published.^4^

To screen for genes required for sexual development, we mutagenized the *P. berghei* reporter line 820,^20^ which expresses green and red fluorescent proteins (GFP and RFP) from promoters specific for male and female gametocytes, respectively (Figure 1A). This line was transfected with pools of barcoded knockout vectors from the *Plasmo*GEM resource^21^ targeting 1,302 genes previously determined to be non-essential for asexual erythrocytic growth (relative growth rate at least half of wild type^16^). Using the expression of fluorescent proteins as proxies for sexual development, parasitized red blood cells from mice infected with each superpool were sorted into male (GFP-positive), female (RFP-positive), and asexual (Hoechst-only) populations (~10^6^ of each; Figure 1B). Barcodes were then counted using barcode sequencing (bar-seq), using amplicons obtained from the genomic DNA of the sorted parasites. After six replicate screens in large pools, 50 top hits were rescreened in duplicate and with a similar number of control mutants. This smaller pool allowed a more precise enumeration of barcodes (see method details for full details and see Table S1 for all screen data).

At the defined significance threshold, 96% of parasite genes that could be queried were not required for the expression of sexual reporter proteins because their respective barcodes were equally represented in the sorted populations (Figure 1B). There were 30 mutants depleted from both sexual populations, another 14 were reduced only in the male, and 21 only from the female population (Figure 1C; Table S1). Reassuringly, hits included the transcriptional activator *ap2-g*^4,6^ and the repressor *ap2-g2*,^4,22^ both known to regulate gene expression during gametocyte formation in *P. berghei*. Other genes affecting both sexes often had weaker effects and were also required for normal asexual growth^16^ (Table S1), suggesting they contributeto cell survivalmore broadly, examples include a putative nicotinamide adenine dinucleotide synthase (PBANKA_0827500) and a pre-mRNA splicing factor (PBANKA_0409100). Only a few mutants resembled *ap2-g* in showing profound effects on both sexual markers, whereas asexual growth was normal. Most notable in this category were a putative ubiquitin-conjugating enzyme (PBANKA_0806000) and a conserved *Plasmodium* protein of unknown function (PBANKA_0824300). The notion that both sexes require these genes for fertility is consistent with evidence from an earlier screen showing that neither the female nor the male gametocyte can pass either of these disrupted alleles to the oocyst stage, which establishes the infection in the mosquito (>24-fold reduction in oocyst numbers shown in the screen of Stanway et al.^18^) (Table S1).

**Figure 2:**
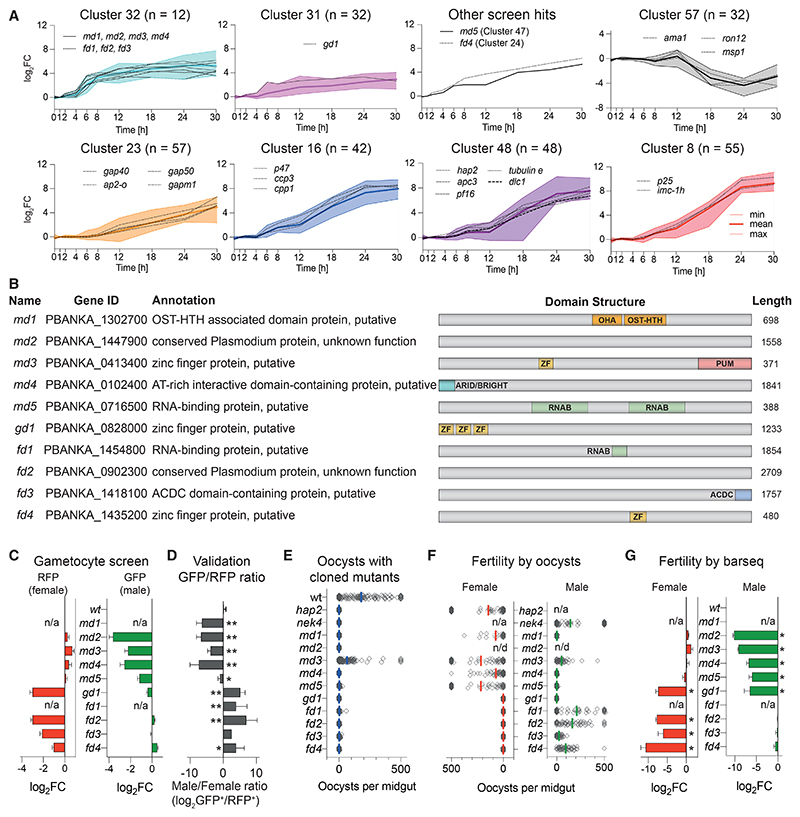
Selection and validation of ten *P. berghei* genes with sex-specific roles in gametocytogenesis (A) Selected gene expression clusters from a bulk RNA-seq time course of induced sexual development. Ring-stage parasites were reprogramed at t = 0 h by inducing *ap2-g*.^6^ Relative transcript abundances are given as log_2_-fold change relative to uninduced, asexually developing parasites. Shown are selected clusters (number of genes) with screen hits designated *md, fd*, or *gd*. Selected well-characterized marker genes of male, female, and asexual development are also shown. (B) Schematic illustration of genes with validated roles in sexual development. OST-HTH, oskar-TDRD5/TDRD7 winged helix-turn-helix domain; OHA, OST-HTH associated domain; ARID/BRIGHT, AT-rich interaction domain; ZN, C3H1 zinc finger; PUM, Pumilio RNA-binding repeat profile; RNAB, RNA-binding domain; ACDC, apetala 2 domain-coincident C-terminal domain; PH-like, PH domain like. (C) Fold-change (FC) in reporter-positive cells in the bar-seq screen. Error bars show standard deviations. (D) Sex ratio in individual mutants determined by flow cytometry. Error bars show standard deviations from 2 to 4 biological replicates with cloned mutants, except for *fd3*, where the uncloned population is shown. * p < 0.05; ** p < 0.01 in unpaired t test. (E) Transmission efficiency of mutant clones *in vivo* determined by counting oocysts on midguts 10 days after an infectious blood meal. (F) Male and female fertility as determined by the ability of mutant clones to give rise to oocysts in mosquitoes when crossed to *nek4* and *hap2* mutants, which provide fertile male or female gametes, respectively. Oocysts counts show combined data from 25 to 80 dissected mosquitoes from 2 to 3 independent experiments. n/d, not done; n/a, not applicable. (G) Fold-change (FC) in female and male fertility determined by bar-seq of 10-day infected midguts following mutagenesis of female-only or male-only lines, respectively. Error bars show standard deviations from four biological replicates. * p < 0.001 in unpaired t test.

Genes with functions specific to a single sex were highly represented among a small group of 60 genes that we previously showed respond within 6 h of experimentally reprogramming ring stages to sexual development by induced expression of AP2-G.^6^ Since early response genes may hold a clue to sex determination, we increased the temporal resolution of the transcriptomic time course to find out which genes respond first. *In vitro*-synchronized schizonts were reprogrammed into gametocytes, injected into mice, and harvested for bulk RNA-seq analysis at additional time points during the first 6 h after induction (Figure S1; Table S2). Co-expression analysis by neural network-based dimensionality reduction now identified an even smaller cluster of 12 co-regulated genes that responded to *ap2-g* induction within 1–2 h and plateaued from 8 to 12 h (Figures 2 and S1; Table S2). This group contained no previously known sex markers, and their transcripts increased before the main wave of around 300 canonical male and female-specific genes, whose expression only began to increase detectably from 8 to 12 h after induction (Figure 2A). This is in contrast to other genes with known roles in gametocytogenesis, which are expressed later during reprogramming (clusters 23, 16, 48, and 8) and genes with functions in the asexual parasite whose transcripts decreased (cluster 57). Single-sex screen hits accounted for five of the early response genes, a significant enrichment (p < 10^−8^, using a one-tailed hypergeometric test). Cluster 32 included several putative nucleic acid-binding proteins of unknown function (Figure 2B), which we hypothesized could be involved either in determining the sex of gametocytes or in their subsequent sex-specific differentiation. To examine this idea further, we selected all five screen hits from this cluster for further validation. We added to the validation group (shown in Figure 2C), three genes from other clusters 24, 31, and 47, which also responded rapidly to *ap2-g* overexpression (Figure 2A). Cluster 32 included two additional genes encoding putative nucleic acid-binding proteins, PBANKA_1302700 and PBANKA_1454800, which had, however, not been covered by the screen because they lacked barcoded *Plasmo*GEM vectors. These genes were selected to complement the hits from the unbiased screen because their domain architecture and expression pattern suggested they may be functionally related. None of the genes in the validation set are required for asexual blood-stage growth according to our earlier screen.^16^

Flow cytometry with individual knockouts in the 820 line confirmed the biased expression of fluorescent sex reporters for all screen hits and further showed sex-specific losses of marker expression for the two newly included early response genes (Figure 2D). Depending on the affected sex, we refer to the validated genes as “male development” (*md1* to *md5*) or “female development” (*fd1* to *fd4*, Figure 2B). As expected, cloned mutants showed a complete or nearly complete loss in their ability to form oocysts in mosquitoes when transmitted individually, with the exception of *md3* in which oocysts were merely reduced (Figure 2E). These experiments demonstrate that the reporter system used in the screen predicts a functional phenotype in transmission. To assess the fertility of each sex individually, we performed genetic crosses of mutants with established maleonly or female-only producing lines using either individual mutants (Figure 2F) or barcoded single-sex pools (Figure 2G). In the first instance, we counted oocysts microscopically, in the second case we counted barcodes (i.e., genome numbers) in infected midgut epithelia as a proxy for fertility. For most genes, fertility was sex-specifically affected precisely as predicted by reporter expression. Two deviations from the screen results were observed. One notable exception was a cloned mutant in PBANKA_0828000, which by FACS only lacked parasites expressing the female marker. However, although gametocytes expressing the male marker were produced, these proved infertile (Figure 2F). Due to its broader gametocyte development (gd) phenotype, we refer to this gene as *gd1*. The second mutant requiring further consideration is *md3*. For this gene, a cloned line was transmitted less well than the wild type (Figure 2E) and males were less fertile in a cross (Figure 2F), but in each case, the effect was less pronounced than the screen result had predicted. By contrast, when the fertility of male mutants was assessed in competition, the transmission of an *md3* mutation was reduced to <1% (Figure 2G), an effect size consistent with a reduction in cells expressing the male reporter gene in the screen. We believe this discrepancy is due to the large excess of zygotes that optimized laboratory infections of *P. berghei* produce. Since oocyst numbers saturate at low input numbers,^23^ we conclude that a competitive experimental design offers a wider dynamic range to measure the relative fertility of a mutant accurately.

### Males and females differentiate from a shared sexual branch

A more precise characterization of the developmental block in each mutant was hampered by the absence of markers that could be used to ask whether gametocytes of the missing sex either failed to form or to differentiate. To address this question, we turned to scRNA-seq. Using the plate-based Smart-seq2 method, we first generated single-cell transcriptomes from 2,028 red blood cells infected with mutant parasites and 689 wild-type controls for comparison. These parasites had all been cultured for 24 h to allow any atypically developing gametocytes to survive without being cleared by the spleen (Figures S2A and S2B).

**Figure 3:**
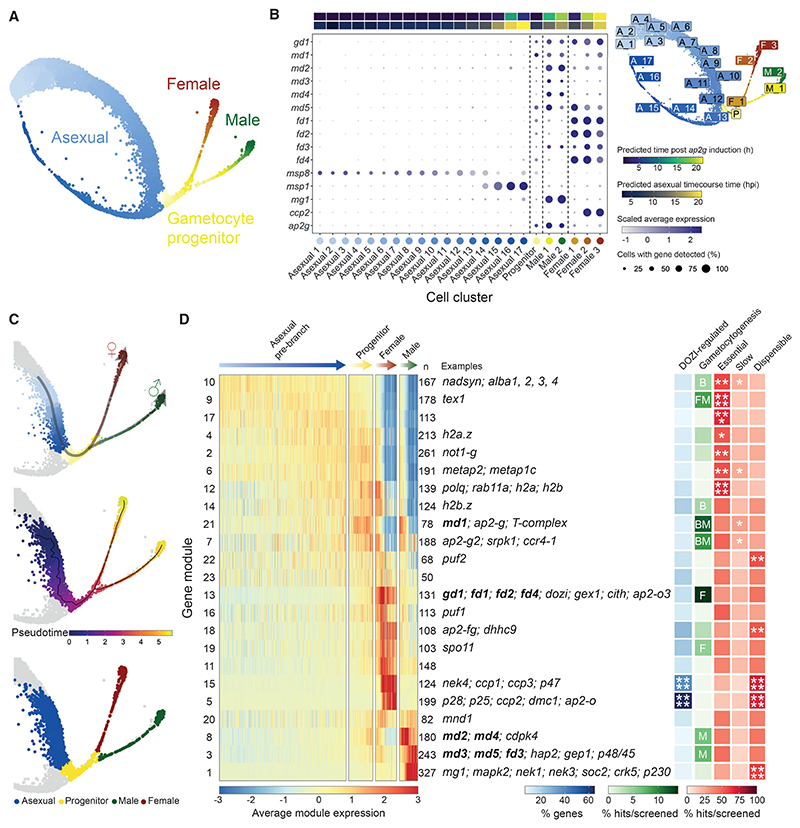
Combined analysis of wild-type 10x and Smart-seq2 transcriptomes from *P. berghei*-infected mouse red blood cells (A) UMAP plot of all 6,880 wild-type cells (Smart-seq2 and 10x), colored by their assigned sex designation and progression along development. (B) Dot plot showing expression of known marker genes alongside the candidate genes in 23 cell clusters. The estimated average time point of each cluster is annotated by correlating single-cell data to bulk time course data (this study and another study conducted by Hoo et al.^24^). Cluster locations are shown on the UMAP plot (top right). (C) Wild-type cells were sub-clustered and 10x-only cells (non-gray dots) were selected in order to re-calculate pseudotime for the branches of interest and construct modules of genes that are co-expressed over pseudotime. These selected cells are colored by sex assignments (bottom), recalculated pseudotime values (middle), and a composite of sex assignments and pseudotime (top), superimposed on the UMAP in (A). Non-branch cells are colored gray. (D) Heatmap showing the scaled average expression of gene modules in cells shown in (C). n, the number of genes per module; DOZI-regulated, % of DOZI-regulated genes within each cluster according to Guerreiro et al.^25^ M, F, and B represent significant (p ≤ 0.05) enrichment of screen phenotype in males only, females only, or both sexes, respectively. Essential, Slow, Dispensable, % genes per cluster with asexual blood-stage phenotype according to Bushell et al.^16^ Significance of enrichment: * p ≤ 0.05; ** p ≤ 0.01; *** p ≤ 0.001; **** p ≤ 0.0001. See Figure 5 for details. Exemplary genes for each cluster are shown.

To better understand gene expression in wild-type parasites, we first removed mutant cells from our dataset and combined all wild-type Smart-seq2 data with a high-resolution map of gametocyte development that we created from 6,191 droplet-based (10x) wild-type single-cell transcriptomes covering the asexual cycle, sexual commitment, and the bifurcation into either sex (Figure S2C). The two wild-type datasets were integrated to generate a combined uniform manifold approximation and projection (UMAP) plot (Figures 3A and S3). Branching and pseudotime analysis on the combined wild-type data showed male and female gametocytes initially follow a common transcriptional trajectory after branching from the asexual cycle before they assume distinct sexual identities (Figures 3A, S3, and S4).

We clustered the wild-type 10x transcriptomes to resolve the branch points of sexual development (Figures 3B and S3). Transcripts from most sexual development genes first became detectable at the joint root of both sexes (Figures 3C, 5, and S4B), where *ap2-g* was also upregulated, but before the transcripts of canonical sex genes such as a dynein heavy chain (*mg1*, male) and *ccp2* (female) became detectable. *md* and *fd* genes were generally upregulated most strongly along the specific sexual trajectory affected by their disruption and thus serve as early sex markers downstream of *ap2-g* (Figure 3B). Having assigned wild-type cells to male, female, and asexual lineages in pseudotime (Figure 3C), we identified co-expression gene modules in the wild-type single-cell data that delineate the sex-specific developmental programs (Figure 3D). Mapping all knockout phenotypes from the screen onto these gene modules shows significant enrichment for gametocytogenesis phenotypes among the first wave of sex-specific genes in each branch (p < 10^−3^ with a hypergeometric test; Figure 3D; Table S3), providing further validation for the screen and independent confirmation of the early response genes first identified in the bulk transcriptomes from the reprogramming time course (Figures 2A and S1).

### Single-cell transcriptomes distinguish differentiation genes from putative sex ratio regulators

**Figure 4:**
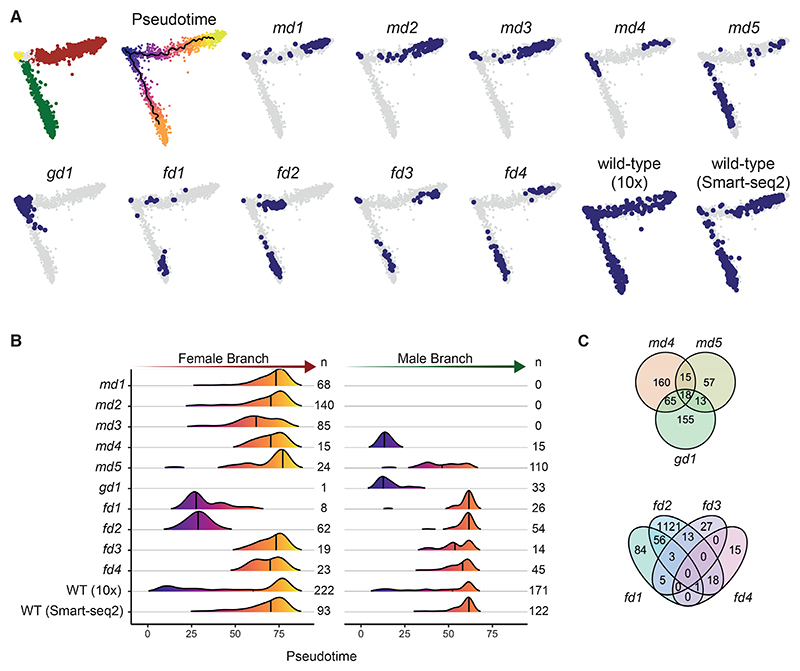
Smart-seq2 analysis of *in vitro-*matured wild-type and mutant parasites (A) Principal-component analysis (PCA) plots of 3,012 transcriptomes from single parasitized red blood cells of the sexual branch that were obtained by merging all wild-type and mutant cells and subsetting the branch of interest (Figure S5). (B) Density plots showing the distribution of assigned female (left) and male (right) cells along each pseudotime trajectory and grouped by genotype. n, the number of cells for each condition as shown in (A). WT, wild type. The line indicates the median. (C) Venn diagrams showing the numbers of genes with differences in transcript abundance in female and male gametocytes, respectively, relative to wild-type. Genes that result in the absence of a sex cannot be evaluated.

To understand the nature of phenotypes from the single-cell data of the mutants, we first investigated their developmental progression in comparison to wild-type parasites (Figures 4A and S5). We then used this integrated dataset to determine the maturation profile of cells for each mutant in each lineage compared with the wild-type reference (Figure 4B). Through this analysis, we observe delays in the normal developmental program of some mutants, but we cannot define unique transcriptomic states associated with aberrant phenotypes. To explore the latter, we created a cellxgene visualization (http://obilab.molbiol.umu.se/gcsko/). Furthermore, we conducted a differential expression analysis of mutants in relation to the wild-type maturation cluster they most correspond to for each sex (i.e., the final state of maturation that each mutant reached) and observed genes that were misregulated in those mutants (Figure 4C; Table S4). We found that differentially expressed genes in mutant parasites were enriched in genes for specific sexual modules identified in Figure 3D (Figure S6; Table S5).

**Figure 5:**
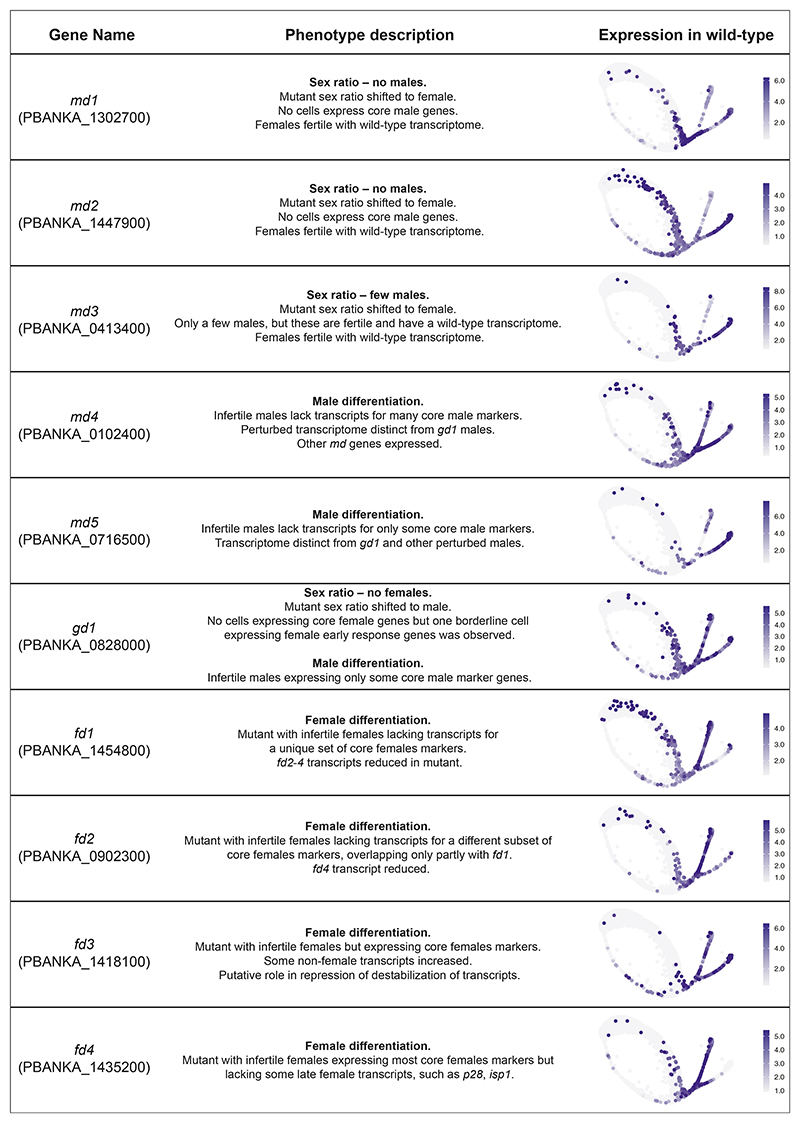
Summary data from Smart-seq2 experiments of mutants A description of the phenotype observed from the single-cell RNA-seq data is given along with a summary of the fertility phenotype (Figures 2E–2G). On the right-hand side, the scaled expression of the gene in wild-type only cells is shown with the 5th and 95th quantiles set as the minimum and maximum expression values, respectively, to eliminate any outliers having a strong influence on visualization.

scRNA-seq showed that mutants fell into two classes: those where cells of the sterile sex were undetectable and those where the sterile sex was still present but had an atypical transcriptional signature (Figures 4A, 4B, and 5). The latter category of mutants still expressed some of the core marker genes of the sterile sex, but cells often clustered separately from the wild type (http://obilab.molbiol.umu.se/gcsko/, Figure S7; Table S4), and more cells resembled earlier points in pseudotime (Figure 4B). This analysis identified *md4, md5, fd1-4* as differentiation mutants because they become committed to a sex but fail to develop their complete transcriptomic signature. While the loss of *gd1* results in the near complete absence of females, it also perturbs differentiation in males (Figures 4A and 4B). Differential gene expression analysis shows each differentiation mutant to be defined by its own transcriptome state in the infertile sex (Figures 4C and S6; Table S4), suggesting each gene exerts its effect in a unique way.

When the sex ratios of mutants were re-assessed using Smart-seq2 data for staging (Figure 4B), four candidate genes for sex ratio determination emerged. *md1* and *md2* are potential maleness-determining genes since their disruption leads to a complete loss of cells expressing male markers. *md3* also showed a marked sex ratio shift toward females. However, consistent with the low-level male fertility retained by this mutant (Figures 2E and 2F), a few *md3* knockout cells expressing the male marker were seen by flow sorting. The transcriptomes of these cells were essentially indistinguishable from wild type (Figure S6), reinforcing the notion that the deletion of *md3* does not affect male differentiation but sex ratio, albeit with less complete penetrance than *md1* and *md2*.

### GD1 is a cytosolic protein that interacts with RNA-binding protein complexes

**Figure 6:**
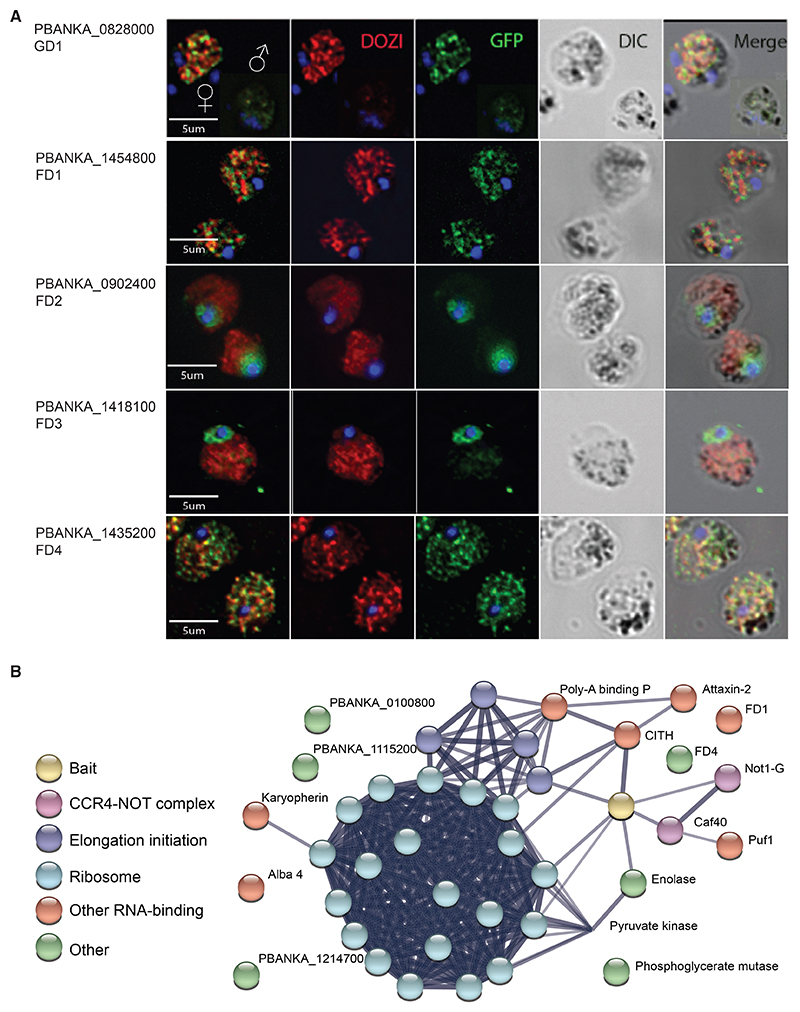
Functional analysis of GD1 and female development genes FD1-4 (A) Immunofluorescence images of fixed gametocytes expressing C-terminally GFP-tagged proteins from their endogenous promoters. Female gametocytes are shown except where indicated. The stress granule helicase DOZI served as a female marker. Images are representative of ca. 500 inspected cells. (B) STRING association network^26^ of selected proteins specifically co-immunoprecipitated with GD1-3xHA with a significance analysis of interactome (SAINT) score probability > 0.9. See Table S6 for a complete list of putative interactors with gene IDs.

The sexual development genes we describe constitute the first wave of genes transcribed within hours when AP2-G is induced. The molecular mechanisms through which they act should therefore be investigated most appropriately in sexually committed ring-stage parasites, which are small and difficult to produce in sufficient quantities for biochemical studies. To nevertheless obtain an indication of the subcellular localization and interaction partners of GD1, we exploited the fact that an endogenously tagged protein was still present 24 h later, in mature gametocytes. The C-terminal addition of a triples haemagglutinin (3xHA) or GFP tag did not interfere with gametocytogenesis and resulted in a protein that was detectable in mature gametocytes by immunofluorescence microscopy and on western blots (Figure 6A, data not shown).

Immunofluorescence microscopy of infected red blood cells in fixed blood films found most GD1 protein in female gametocytes, with less staining in males and no staining in asexual blood-stage parasites (Figure 6A). GD1 staining produced a punctate cytosolic pattern that was of similar granularity as that of the mRNA binding protein DOZI, a DDX6 class RNA helicase that we chose as a marker for female gametocytes. However, GD1 and DOZI were largely not overlapping.

Immunoprecipitates of GD1-3xHA from mixed blood stages parasites were analyzed by protein mass spectrometry and found to be enriched in proteins associated with mRNA binding and processing functions when compared with control pull-downs from cells in which GD1 was not tagged (Figure 6B; Table S6). Putative GD1 interactors include members of the CCR4-Not complex, including the gametocyte-specific Not paralog, Not1-G, and as minor components DOZI, PUF1, and a putative mRNA decapping enzyme.

Additionally, GD1 precipitated the female differentiation factors FD1 and FD4 (Figure 6B; Table S6). Consistent with these data, epitope-tagged FD1 (a putative mRNA helicase) and FD4 (another putative zinc-finger [ZNF] protein) gave rise to a GD1-like speckled cytosolic immunofluorescence pattern in female gametocytes (Figure 6A). By marked contrast, epitope-tagged FD2 and FD3 proteins did not co-immunoprecipitate with GD1 and appeared nuclear by immunofluorescence microscopy of tagged proteins. Taken together, these data point to important roles for posttranscriptional mechanisms in the regulation of sex determination and differentiation in a malaria parasite.

## Discussion

Through an unbiased functional screen, we have identified 65 genes with functions essential for gametocytogenesis in *P. berghei*, many of which are unannotated and unstudied. Using a reporter system of sex-specific promoters that turn on around 8 h after induction, we have targeted the screen to the first few hours of sexual development to find genes required for the determination of sex ratio and early during the subsequent emergence of sex-specific programs of gene expression.

Although this screen constitutes significant progress toward gaining a functional understanding of early sexual development in a malaria parasite, we do not claim it to be comprehensive but recognize three major limitations. First, genes that are essential in asexual blood stages were not included in the screen, and any additional functions that such genes have in gametocytogenesis can only be revealed through conditional knockout approaches. Interestingly, the category of asexual lethal genes hypothetically includes suppressors of sexual development, i.e., genes whose disruption would be sufficient to switch parasites to a sexual path. Second, the *Plasmo*GEM resource is incomplete, covering only around 65% of protein-coding genes. We are currently unable to remedy this limitation because some genes fail at vector production, whereas for others, vectors integrate poorly for unknown reasons. However, we demonstrate here how screen results can be used to predict phenotypes of mutants that the screen did not cover, and we include two such genes in our validation set. Third, the proxy phenotypes provided by reporter cassettes need to be interpreted with caution. Although our validation experiments clearly show that the screen succeeded in discovering genes affecting development and differentiation, we also found mutants where fewer gametocytes coincide with slower asexual growth, suggesting the underlying gene function is not sex specific. Furthermore, since the screen was conducted *in vivo*, gametocyte mutants with normal development but altered extravasation, de-sequestration, or enhanced splenic clearance may be among the hits, and secondary screens could be envisaged to identify such interesting mutants from among the genes we report here.

We chose to focus our follow-up experiments on 10 sexual development genes whose transcripts are enhanced in the first wave immediately after reprogramming gametocytes by induced AP2-G expression. Half of these genes bind AP2-G in their promoters in mature gametocytes.^27^ All have sex-specific functions for the production of mature gametocytes, which we show to be critical for malaria parasite transmission to the vector. Using single-cell analysis of mutants we characterize the unique role of each gene in sex ratio determination and subsequent cellular differentiation. A summary of data for all genes can be found in Figure 5.

**Figure 7:**
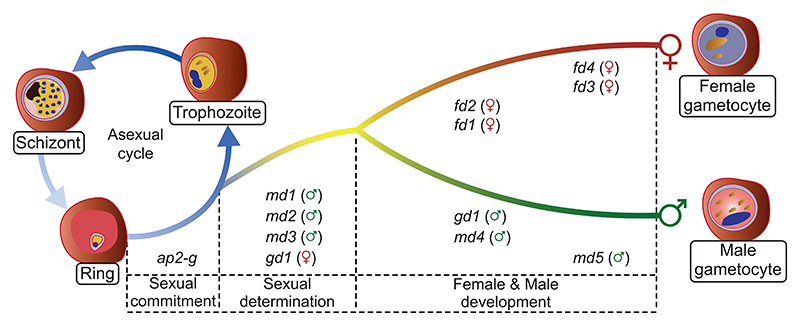
Model of how sexual determination and differentiation is affected by a cascade dominated by putative nucleic acid-binding proteins identified in this study In addition to its role in commitment, *ap2g* probably has sex-specific roles in differentiation,^27^ together withthe female-specific transcription factor AP2-FG, which binds in the promoter regions of *fd2* and *fd4*.^13^

Our functional and transcriptomic data suggest a model in which male and female *P. berghei* gametocytes differentiate from a common sexual precursor in ways that rely on a cascade of nucleic acid-binding proteins that are co-expressed downstream of AP2-G and fulfill distinct functions in a hierarchy of regulatory events (Figure 7). In this hierarchy, the triple ZNF protein GD1 is a candidate for a top-level factor for female determination or differentiation upstream of the female-specific transcription factor AP2-FG.^13^ That GD1 co-immunoprecipitates a broad range of mRNA-interacting proteins suggests GD1 may itself interact either directly or indirectly with mRNA molecules and contribute to regulating their stability or translation. However, although these data provide a hint as to potential mechanisms, they need to be interpreted with caution. Among the mixed blood-stage parasites from which GD1 was precipitated, immunofluorescence analysis indicates it was the mature female gametocytes that contributed most of the GD1 protein. We do not know if GD1 has a function in maturing female gametocytes since loss-of-function mutants are blocked around 24 h earlier before cells with a female transcriptome become detectable. To get at the mechanisms underpinning the early functions of the genes our screen identified, it will be necessary to identify their interacting proteins or nucleic acids from synchronously developing parasites immediately after commitment to sexual development. This is a technical challenge we have not yet solved. It is also important to consider that the interactors identified in the current study may be direct binding partners or they may reflect a broader assemblage of proteins connected through the mRNA molecules they interact with. Notwith-standing these caveats, it is interesting to note that FD4 and the putative RNA helicase FD1 may in some way connect with GD1 and that they have a similar specked distribution in the cytosol of mature female gametocytes that is typical of mRNA-regulating stress granules or P-bodies.

Among the female differentiation genes, *fd1* encodes a putative RNA-binding protein, and *fd2* encodes a conserved *Plasmodium* protein. Both have profoundly perturbed female transcriptomes, and *fd1* mutant females expressed only some female markers. By contrast, *fd3* and *fd4* have more subtle roles in the formation of transcriptionally normal females. *Fd3* is characterized by an AP2-coincident C-terminal domain of the unknown function (apetala 2 domain-coincident C-terminal domain [ACDC]). This type of domain was first identified in a number of *P. Falciparum* AP2 domain-containing proteins,^28^ but we found FD3 is localized in the nucleus, confirming another recent report that found this protein is required for female fertility.^27^

*Fd4* encodes another putative ZNF protein localizes to both the cytosol in mature females (Figure 6A). The mutant only shows a moderate downregulation of a few late female transcripts (Figures 4C and S7; Table S4). *Fd4* is one of the transcripts that are less abundant in *fd1* and *fd2* mutants (whereas the reverse is not the case), suggesting the latter genes may operate upstream of *fd4*. Taken together, our data suggest female differentiation is regulated by a cascade of nucleic acid-binding proteins that are co-expressed downstream of AP2-G and fulfill distinct functions in a hierarchy of regulatory events.

*md1* is the most noticeable AP2-G responsive gene to be up-regulated at the base of the sexual branch (Figure 3D). It encodes a protein with a putative domain named alternatively LOTUS (after Limkain, Oskar, and Tudor domain-containing proteins 5 and 7) or OST-HTH (for oskar-TDRD5/TDRD7 winged helix-turn-helix).^29,30^ OST-HTH domain proteins exist in pro- and eu-karyotes, but only those of animals have been studied in detail and all are involved in gametogenesis.^31^ Examples include Os-kar, an important germ line determining factor in *Drosophila* embryos^32^ and the tudor domain-containing proteins (TDRD5 and TDRD7) with sex-specific fertility functions in mice.^33^ LOTUS domains have been proposed to serve as scaffolds for ribonucleoprotein networks within P granules that help recruit and balance essential RNA processing machinery to regulate key developmental transitions in the germ line.^34^ It may therefore be significant that the ZNF gene *md3* contains a weak homology with a pumilio RNA-binding domain, while *md2* lacks clear homologs outside of *Plasmodium*. Together, these considerations raise the intriguing possibility that creating the male lineage in *P. berghei* shares elements with germline definition in multicellular organisms.

Male differentiation mutants are *gd1, md4*, and *md5* in order of decreasing severity with respect to the presence of core male transcripts (Figure S7). Disrupting *gd1* and *md4* has profound but distinct effects on male gene expression. *md4* encodes a conserved *Plasmodium* protein of unknown function characterized by a putative N-terminal ARID/BRIGHT DNA-binding domain, which in other eukaryotes targets developmental transcription factors to AT-rich DNA sequences,^35^ suggesting it may regulate transcription downstream of the initial commitment to the male developmental trajectory. Transcript abundance of *md4* decreases in mature males, but it stays high in *gd1* mutants (Figure S7), illustrating the early developmental arrest of *gd1* males and also indicating that *gd1* is not required for the expression of *md4*. The deletion of *md5* has a less severe effect on core male transcripts and likely operates through a different mechanism because the protein contains putative RNA-binding motifs and localizes to both the nucleus and the cytoplasm (Figure 6A).

All ten *P. berghei* genes validated here have orthologs in *P. falciparum* (Table S7), where their transcripts are all upregulated during sexual development, with seven transcripts peaking during early (stage I–II) gametocytogenesis.^36^ Furthermore, the chromatin immunoprecipitation of AP2-G in synchronously developing parasites identified *P. falciparum* orthologs of *gd1, fd1, fd2, md3*, and *md4* as likely direct targets for AP2-G binding in stage I gametocytes but not in sexually committed ring stages or asexual schizonts.^37^ Further validation of the screen comes from recent functional analyses of three of these proteins in *P. falciparum*. In each case, the analysis of the ortholog shows broad conservation in gene function, although there are notable species differences, possibly reflecting the unique cell biology and slower maturation of *P. falciparum* gametocytes. The putative RNA-binding protein FD1 was described in *P. falciparum* as macrogamete-contributed factor essential for transmission (*Pf*MaCFET), which, like FD1, localizes to cytosolic granules, suggesting the female-specific role of this protein may be broadly conserved in *Plasmodium*.^38^ The MD4 ortholog *Pf*ARID was found to be a nuclear protein, which, like MD4 has an essential role in establishing a fully developed male transcriptome and microgametocytes capable of exflagellation, but unlike MD4, the *P. falciparum* ortholog makes an additional albeit possibly later contribution to female fertility.^39^ Finally, the ZNF protein GD1 has a *P. falciparum* ortholog, ZNF4, which also functions in gametocytes but affects mainly male development and transcripts.^40^ Taken together, these data indicate that the first steps of gametocyte differentiation following the initial commitment to sexual development involve a very similar set of players in different malaria species but that the detailed roles they play may differ in some cases.

Additional work is required to identify how the sex ratio is determined in *P. berghei*. One view of how the sexes are formed in *P. falciparum* is that commitment to a particular sex coincides with or even precedes the induction of sexual development by AP2-G.^41^ In that case, gene expression in response to AP2-G would be expected to follow a sex-specific pattern from the start. Importantly, neither our global transcriptomic analysis of single cells nor the expression of candidate genes from the functional screen produced evidence that a sex-specific transcriptomic signature precedes the commitment to sexual development. Instead, we observed a common branch of sexual precursors and discovered key roles for AP2-G early response genes, suggesting sex may be determined downstream of *ap2-g* induction.

Alternatively or additionally, non-transcriptional mechanisms may operate upstream of *md1-3* and *gd1*, involving, for instance, chromatin marks, differential splicing, or phosphorylation states that determine the emergence of sex-specific transcriptional signatures downstream of AP2-G.

Notwithstanding its early role in the switch to sexual development, AP2-G also binds to the upstream sequences of many male- and female-specific genes later during gametocytogenesis.^27^ Future research will need to determine if either the binding of AP2-G or its activity as a transcriptional activator for these genes is specific to a particular sex and how it is regulated by additional factors.

In summary, our functional screen, in combination with single-cell transcriptomes of cloned mutants, has identified a diverse group of proteins that are co-expressed downstream of AP2-G and whose deletion affects either the determination of or differentiation along a male or female cellular trajectory. Further analysis of these genes will shed light on the precise molecular mechanisms of sex ratio determination and sexual development. The screen revealed an abundance of putative RNA-binding proteins, including the maleness-inducing factor MD1 and the early response genes MD4, MD5, and FD1, whose molecular targets now need to be identified. The LOTUS/OST-HTH domain gene *md1* raises the intriguing possibility that the function of this domain in gametogenesis is conserved beyond animals, possibly to the origin of sex in the ancestral eukaryote. Sex determination mechanisms evolve rapidly but often involve RNA-dependent regulation, for instance, through differential splicing or translational repression,^42–44^ and our data suggest similar principles operate in *P. berghei*. Discovering the molecular targets and interactors of the proteins of GD1, MD1, and FD1 now provide a route to establishing these mechanisms in more detail.

## STAR+METHODS

Detailed methods are provided in the online version of this paper and include the following:

- KEY RESOURCES TABLE
- RESOURCE AVAILABILITY
  ○ Lead contact
  ○ Materials availability
  ○ Data and code availability
- EXPERIMENTAL MODEL AND SUBJECT DETAILS
  ○ Parasite lines
  ○ Use of rodents
  ○ Generation of mutant superpool
- METHOD DETAILS
  ○ Screening of mutant superpool
  ○ Targeted screen
  ○ Screen analysis
  ○ Gene targeting vectors and primers
  ○ Generation of recombinant *P. berghei* with specific genes tagged with **HA** or GFP
  ○ Single **knockout** transfections and genotyping
  ○ **FACS** of single gene ko lines
  ○ Mosquito infections and genetic crosses
  ○ Bulk-**RNA-seq**
  ○ Smart-seq2 scRNA-seq
  ○ 10x Genomics chromium scRNA-seq
  ○ Bulk **RNA-seq data** analysis
  ○ Mapping and generation of expression matrices for scRNA-**seq data**
  ○ Filtering and normalization of scRNA-**seq data**
  ○ **Single-cell** transcriptome analysis of wild-type data
  ○ **Single-cell** transcriptome analysis of single knockout mutant **data**
  ○ Immunofluorescence microscopy
  ○ **Immunoprecipitation** and **mass spectrometry analysis**
  ○ Fractionation of *Plasmodium* proteins into cytoplasmic, nuclear and nuclear insoluble extracts
- QUANTIFICATION AND STATISTICAL ANALYSIS
- ADDITIONAL RESOURCES

## Star+Methods

### Key Resources Table

**Table:** 

REAGENT or RESOURCE	SOURCE	IDENTIFIER
Antibodies		
Rabbit anti-DOZI	This manuscript	N/A
chicken anti-GFP	Abcam	Cat#ab13970
goat anti-rabbit Alexafluor 594	ThermoFisher	A-11012
goat anti-chicken Alexafluor 488	ThermoFisher	A-11035
Critical commercial assays		
10x Genomics Chromium 3’ v2	10X Genomics	PN-120237
BD Influx cell sorter	BD	N/A
Deposited data		
Single-cell RNA-seq data	This manuscript	ENA: PRJEB44892
Bulk RNA-seq data	This manuscript	GEO: GSE110201, GSE168817
Code	This manuscript	https://doi.org/10.5281/zenodo.7317469
mass spectrometry proteomics data	This manuscript	PRIDE: PXD033827
Experimental models: Organisms/strains		
*P. berghei*: 820cl1m1cl1 line parasites	Mair et al.^20^ and Ponzi et al.^45^	820cl1m1cl1
*P. berghei*: PB_GAMi_ line parasites	Kent et al.^6^	PB_GAMi_
Rat: RCC Han Wistar outbred (female)	Envigo	RccHan:WIST
Mouse: BALB/c inbred (female)	Wellcome Sanger Institute & Envigo & Charles River Europe	BALB/cOlaHsd
Mouse: SCID (female)	Envigo	C.B-17/IcrHan®Hsd-*Prkdc^scid^*
Mouse: outbred TO (female)	Envigo	HsdOla:TO
Mouse: C57BL/6N (female)	Envigo	C57BL/6N
Oligonucleotides		
PCR primers used in this study	This manuscript	itemized in Table S1
Recombinant DNA		
Gene targeting vectors used in this study	https://plasmogem.umu.se/pbgem/	Itemized in Table S1
Software and algorithms		
Rstudio (v4.0.3)	https://www.rstudio.com/	N/A
GraphPad Prism 9	http://www.graphpad.com/scientific-software/	N/A
R (v4.0.3)	https://www.r-project.org/	N/A
Flowjo (v7.6.5 and 10.6.1)	https://www.flowjo.com/solutions/flowjo	N/A
Samtools (v.2)		N/A
HISAT2 (v2.1.0)	Kim et al.^46^	N/A
HT-seq (v1.3.1 and v0.11.2)	Anders et al.^47^	N/A
DESeq2 (v1.18)	Love et al.^48^	N/A
Cell Ranger (v2.1.1)	10X Genomics	N/A
Seurat (v3.2.2)	Stuart et al.^49^	N/A
Mixtools (v1.2.0)	Benaglia et al.^50^	N/A
Monocle 3 (v0.2.3.0)	Cao et al.,^51^ Qiu et al.,^52^ and Trapnell et al.^53^	N/A
UMAP	McInnes et al.^54^	N/A
Discoverer v. 2.4	Thermo Fisher	N/A
SAINTexpress	Teo et al.^55^	N/A

## Resource Availability

### Lead contact

Further information and requests for resources and reagents should be directed to and will be fulfilled by the lead contact, Oliver Billker (oliver.billker@umu.se).

## Materials availability

Parasite lines used in this study are freely available under a material transfer agreement for not-for-profit research and should be requested directly from the PlasmoGEM resource (https://plasmogem.umu.se/pbgem/).

## Experimental Model And Subject Details

### Parasite lines

The mutants assayed in the the barseq screen and phenotyped by FACS, in mosquito feeds or scRNA-seq experiments as dilution cloned single gene knockout lines were generated in the *P. berghei* 820cl1m1cl1 line (referred to as 820) that expresses GFP under the control of a male-gametocyte-specific promoter and RFP under the control of a female-gametocyte-specific promoter.^20,45^

### Use of rodents

All animal research at the Wellcome Sanger Institute was conducted under licenses from the UK Home Office, and protocols were approved by the Animal Welfare and Ethical Review Body of the Wellcome Sanger Institute. Rats were housed as two cage companions and mice as five cage companions. They were housed in individually ventilated cages (IVC) furnished with autoclaved aspen woodchip, fun tunnel and Nestlets at 21 ± 2°C under a 12:12 hr light dark cycle at a relative humidity of 55 ± 10%. Rodents were kept in specific-pathogen-free conditions and subjected to regular pathogen monitoring by sentinel screening.

Female RCC Han Wistar outbred rats (Envigo, UK) aged seven to sixteen weeks were infected with *P. berghei* parasites by intraperitoneal injection. Infected rats served as donors for *ex vivo* schizont cultures typically on day four to five of infection, at a parasitemia of ~1%–5%. Rats were terminally anaesthetized by vaporized isoflurane administered by inhalation prior to terminal bleed. Rats were used because they give rise to more schizonts with higher transfection efficiency compared to mice. Transfection efficiency is critical when screening pools of vectors.

Mice used at the Wellcome Sanger Institute were bred in-house or purchased from Envigo, UK. To generate barseq pools and superpools transfected parasites were injected intravenously into the tail of female adult BALB/c inbred mice aged six to eleven weeks. There is one exception, which is that some pools of slow mutants were grown in seven week old female SCID (Prkdc^scid^) mice. The Balb/c animal model was chosen to minimize host genetic variability and to obtain robust infections with a low incidence of cerebral pathology. SCID mice were selected due to their lack of adaptive immunity to improve the representation of slow mutants that are commonly lost during passage. Generation of single gene ko lines, revival of frozen stabilates and dilution cloning was carried out in either adult female BALB/c mice, or adult female outbred TO (HsdOla:TO) mice, which also generate robust infection with low rates of cerebral malaria. FACS analysis and scRNA-seq analysis of single gene ko lines was performed in female BALB/c aged eight to fourteen weeks. Genetic crossing experiments and mosquito feeds were performed using female TO mice and backbites using female C57BL/6N mice. C57BL/6N mice were used for backbites as they are the most susceptible to malaria infection by sporozoites.

The animal research at Umeå University was conducted under Ethics Permit A13-2019 and approved by the Swedish Board of Agriculture (Jordbruksverket). Mice were group-housed as four cage companions in IVC with autoclaved woodchip, paper towels for nesting and a paper fun tunnel or kidney dish, at 21 ± 1°C under a 12:12 h light dark cycle at a relative humidity of 55%.± 5%. Specific-pathogen-free conditions are maintained and subjected to Exhaust Air Dust (EAD) monitoring and analysis biannually. The mice used at Umeå University were purchased from Charles River Europe. Female Balb/c aged 6-20 weeks old were used to bring up stabliates, carry out dilution cloning, do mosquito feeds and to perform FACS analysis of single gene ko lines.

All animal work in Glasgow was approved by the University’s Animal Welfare and Ethical Review Body and by the UK’s Home Office (PPL 60/4443). The animal care and use protocol complied with the UK Animals (Scientific Procedures) Act 1986 as amended in 2012 and with European Directive 2010/63/EU on the Protection of Animals Used for Scientific Purposes. Mice in Glasgow facilities are held in groups of up to 5 per cage, in IVC, containing standard woodchip/aspen, sizzle nesting, fun tunnels and dome homes. Room temp is 21.5 ± 1°C with a 12:12 light dark cycle and a relative humidity of 50% ± 6%. Specific-pathogen-free conditions are maintained and subjected to analysis annually. Parasites were maintained in Theiler’s original (TO) or NIH Swiss outbred female mice, approximately weighing 25 g and > 6 weeks old.

Animals at all sites were fed a commercially prepared autoclaved dry rodent diet and water, both available *ad libitum*. The health of animals was monitored by routine daily visual health checks. The parasitemia of infected animals was determined by methanol-fixed and Giemsa-stained thin blood smears.

### Generation of mutant superpool

Gene targeting vectors used in the barseq screen were obtained from the *Plasmo*GEM resource (https://plasmogem.umu.se/pbgem/),^21^ from where details of vector designs and sequences of gene-specific primers are also available. The barseq screening vectors carried the default *Plasmo*GEM 3xHA-hdhfr-yfcu gene replacement cassette,^56^ and are itemised in Table S1.

To allow genome-scale phenotyping in a single experiment by assaying of all mutants in a single mouse we created superpools of all viable *P. berghei* mutants using pools of vectors matched for integration efficiency and asexual fitness in.^16^ All genes identified as dispensable in the asexual screen were allocated into 9 groups based on their normalised abundance on day 6 of the infection, which was taken to represent vector integration efficiency. Genes giving slow asexual growth in the asexual screen were allocated into three groups based on their relative growth rate, and then each of these groups was further divided into two based on normalised abundance on day 6. This gave a total of 9 pools of dispensable genes (Pool 1-9: 100-110 vectors per pool) and 6 pools of genes generating slow-growing mutants (Pool 10-15: 65-70 vectors per pool). Vectors were picked for all pools and prepared in a pooled midi-prep approach as discussed previously.^16^

All pools were transfected individually into rat-derived 820 *P. berghei* schizonts and injected intravenously (IV) into mice. Transfectants were selected by 0.07 mg/mL pyrimethamine administered in drinking water, all as previously described.^16,57^At a parasitaemia of 1-5% (day six to seven post-trans infection for dispensable mutant pools 1-9, and day seven to nine for slow mutant pools 10-15) infected blood was collected and frozen down.

## Method Details

### Screening of mutant superpool

Stabilates of mutant pools were thawed and combined into different superpools of normal and slow growing parasites such that each vector was part of four independent screening experiments. Figure 1A), and then immediately injected IV into mice and propagated under pyrimethamine selection. On day 6-7 of the infection (parasitaemia ~10%), a sample of (~100 μL) infected blood was collected as “input” and the rest of the blood was collected directly into 4 mL gametocyte non-activation medium (RPMI1640 with L-glutamine, without phenol red and sodium bicarbonate (Sigma) supplemented with 0.1% BSA, 4mM sodium bicarbonate and 20 mM Hepes at a pH of 7.25) at room temperature. White blood cells were removed by passing through Plasmodipur filters (Proxima) and gametocytes were subsequently purified on a Histodenz (Sigma) density gradient (13.25% w/v). Following purification, parasites were washed and stained with Hoechst 33342 (Thermo Fisher) prior to sorting. Throughout handling, care was taken to avoid gametocyte activation by not exposing infected blood or parasite pellets to air, performing all steps in the gametocyte non-activation medium and keeping all reagents at room temperature.

GFP+ Hoechst+ (male gametocytes), RFP+ Hoechst+ (female gametocytes) and Hoechst+ only (asexual parasites) populations were isolated and sorted using a BD Influx cell sorter. Typically 0.5-1.0 x10^6^ cells were collected for the GFP^+^ Hoechst^+^ and RFP^+^ Hoechst^+^ populations and 1.0-2.0 x10^6^ cells were collected for the Hoechst+ populations from each sort. Following sorting, cells were collected by spinning at 2000 x *g* for 10 min,.in 5 mL Eppendorf tubes, supernatant was carefully removed and pellets frozen at -20 °C. For the input infected blood sample, erythrocytes were lysed using NH_4_Cl and parasites were pelleted by centrifugation; the supernatant was removed and pellets were frozen at -20°C. Genomic DNA was extracted from the pellets upon thawing using phenol-chloroform. gDNA from sorted gametocyte populations was reconstituted in 10 μL dH_2_O. For input infected blood sample, gDNA was dissolved in 100 μL. 5 μL gDNA was used as input for the first PCR reaction when generating sequencing libraries, with each reaction run in duplicate. Barcode sequencing libraries were generated using a nested, direct-amplification PCR approach with Illumina index tag primers to allow multiplexing of all samples from one experiment. Libraries were sequenced on a MiSeq (Illumina) at cluster density of 400 K with 50% PhiX spiked-in, all as previously described.^17^

### Targeted screen

The screen was adapted to re-screen all gametocyte phenotype hits from the initial genome-scale screen (forming the “targeted screen”). This targeted screen pool contained <166 mutants (Table S1) and its mutant pools were generated by transfecting two pools of 83 constructs into two separate mice as described as above. Resulting stabilates were mixed together and re-injected into mice. For the targeted screen conducted *in vivo*, the experiment was performed exactly as outlined above. Gametocytes were purified and sorted with mutant barcodes sequenced as before.

### Screen analysis

Barcodes in Illumina sequencing data were counted and tabulated per library. To facilitate downstream analysis, these counts were expressed as log_2_ proportions, with 0.5 added to counts. To quantify the uncertainty induced by the fact that the barcodes that successfully entered libraries were a random sample of those in the actual population (with sampling occurring at the time of purification, sorting, pipetting of template material, and during amplification), standard deviations were calculated for each barcode in each sample, based on technical PCR duplicates. These standard deviations were made more precise by taking the moving average of values (k=11), with samples ordered by the abundance of the barcode in question, and enforcing monotonicity. This approach provided an estimate of the log_2_ proportion made up by each barcode in each sample, with an associated uncertainty estimate. For each pool analysed, results were available based on sorted populations derived from three mice. We calculated a fold-enrichment for each sample of interest (the fluorescently sorted populations) as compared to the negative control (the Histodenz gradient for super pool 3 (SP3), and specifically sorted non-fluorescent parasites for super pool 4 (SP4) & the targeted screen super pool 6 (SP6)), and propagated uncertainties to this value. To normalise these values within each sample, we calculated an estimate for the change in abundance of seven control genes (PBANKA_071830, PBANKA_120780, PBANKA_083110, PBANKA_051060, PBANKA_051820 & PBANKA_132610), and computed an inverse-variance weighted mean for the change in this control sample (all inverse-variance weighted mean calculations used the method described in Bushell et al.^16^). We then normalised by subtracting this control value from each value of interest. To yield a combined estimate across all mice and experiments, we calculated the inverse-variance weighted mean, and its expected uncertainty using the method described in Bushell.^16^ Named phenotypes were assigned using thresholds based on the upper and lower bounds of the confidence intervals for enrichment in the GFP-positive and RFP-positive populations: if the confidence interval overlapped a two-fold reduction in barcode counts, we considered that we did not have power to detect a substantial reduction (*no power*), if the confidence interval lay entirely with a reduction of more than two-fold, we considered this a significant reduction (*reduced*) and if the confidence interval lay entirely with a reduction of less than two fold, we considered this to represent no substantial reduction.

### Gene targeting vectors and primers

To disrupt genes individually, *Plasmo*GEM vectors were used for *md2, md3, md4, md5, gd1, fd2, fd3* and *fd4* (Table S1). Since *Plasmo*GEM vectors were not available to target *md1 and fd1*, knockout vectors were prepared using PCR. For *md1*, amplicons of 1.25 kb upstream and downstream of the gene were amplified from genomic DNA and then assembled either side of a selection cassette amplified from the *Plasmo*GEM 3xHA-hdhfr-yfcu gateway vector^56^ by Gibson Assembly using primer overhangs. The Gibson product was used as the input for a PCR reaction to amplify the entire construct, which was then gel-purified prior to transfection.

For *fd1* a CRISPR/Cas9 knockout vector was constructed by PCR amplification of 0.5 kb 5’ and 3’ regions of the coding sequence of the target gene from genomic DNA, which were restriction-ligation cloned so to flank an *eef1a* 5’UTR-*tgdhfr-CAM* 3’UTR resistance cassette in a vector also holding the U6 RNA Polymerase 3 promoter from *Plasmodium yoelii* to drive expression of the target specific guide RNA (gRNA) cloned in by BsmBI, to generate plasmid ABR063. The fd1 CRISPR/Cas9 knockout line was generated in a split-Cas9 line where conditional activation of CAS9 is achieved by rapamycin-induced dimerisation of a C-terminal fragment of Cas9 fused to FK506- and rapamycin-Binding Protein domain (C-Cas9-FKBP) and a N-terminal fragment of Cas9 fused to the FKBP-Rapamycin Binding domain (N-Cas9-FRB). To achieve this line, N-Cas9-FRB and C-Cas9-FKBP was cloned so to flank the bidirectional 5’UTR of eef1α, and to become nested within 5’ and 3’ targeting sites for the p230p locus generating plasmid ABR010. Primer sequences for cloning custom knockout vectors and genotyping the resulting parasites are displayed in Table S1.

For scRNA sequencing experiments, where possible, FACS sortable knockout vectors were generated by converting *Plasmo*GEM intermediate vectors^17^ into knockout vector with a gateway cassette containing an pbhsp70 5’utr-GFPmut3-pbdhfr 3’utr-hdhfr-yf-cu.expression cassette for constitutive expression of GFP upon vector integration, which allows for FACS sorting to select wild-type free ko parasites without the need for dilution cloning. The gateway construct was generated by amplifying a 1.4 kb fragment of the 5’utr of hsp70 from *P. berghei* genomic DNA, which was cloned into the R6K-GFPmut3-hdhfr-yfcu Gateway vector (added to the *Plasmo*GEM tagging vector repertoire described in Gomes et al.,^17^ upstream of GFPmut3 to generate the R6K-hsp70p-GFPmut3-hdhfr-yfcu Gateway vector. When no FACS-sortable knockout vector could be generated in this way, scRNA-seq analysis was performed on dilution cloned mutants generated using the standard *Plasmo*GEM knockout vectors harbouring the 3xHA-hdhfr-yfcu cassette (*md2* and *md3*) or custom knockout vectors (*md1* and *fd1*).

### Generation of recombinant *P. berghei* with specific genes tagged with HA or GFP

C-terminal tagging of GD1 for microscopic analysis used a *Plasmo*GEM vector, while FD1, 2, 3 and 4 were c-terminally tagged with 3xHA and GFP, and GD1 with GFP using a single cross-over mechanisms with plasmids derived from pG548 for GFP and G0201 for HA. For these mutants, vector names and oligos used for their generation and PCR verification of integration are given in Table S1. Regions of homology lacking a stop codon for SCO were generated by PCR using the primers indicated, incorporating a 5’ NotI site and a 3’ XhoI site and maintaining the open reading frame. Linearisation of the vector within the coding region of the target gene was achieved either through using a natural unique restriction site or through incorporation of an Eco RV site amplifying the region of homology in two fragments which were then joined by sequential cloning into the tagging vector. Further details are given in Philip et al.^58^ For protein pull-down experiments, GD1 was fused c-terminally with 3xHA epitope tag using a CRISPR strategy. Plasmid Pb_MH_052 was used to tag GD1 c-terminally by transfecting a line constitutively expressing a rapamycin-dimerizable Cre recombinase. The recombinase was not used in the current study, but the background was chosen to enable sexual reprogramming in future experiments.

### Single knockout transfections and genotyping

To disrupt genes individually, *Plasmo*GEM vectors were used for *md2, md3, md4, md5, gd1, fd2, fd3* and *fd4* (Table S1). Vectors were prepared using QIAGEN Plasmid Midi Kit and 1-5 ug NotI digested and ethanol precipitation purified DNA was transfected into *P. berghei* 820 rat-derived schizonts. Transfected parasites were injected intravenously into mice and selected using pyrimethamine for pooled transfections above. For *md1* and *fd1*, 1 μg of custom vector DNA was transfected into schizonts as above, with the single modification that mouse-derived schizonts were used.

### FACS of single gene ko lines

FACS was performed either on parasites revived from frozen stabilates (typically on day 5 post-injection at a parasitaemia of >2 %).or direct from transfection (typically on day 7-8 post-transfection at a parasitaemia of >2 %). Gametocytes were analysed directly from infected mouse blood or were purified from using gametocyte non-activation medium and Histodenz density gradient, and stained with Hoechst 33342 as above. Purified and stained gametocytes were resuspended in gametocyte non-activation medium, taking care to not expose parasite pellets to air and kept at room temperature at all times. Samples were immediately analysed for mCherry (female gametocytes) and GFP (males gametocytes) using a BD LSR Fortessa instrument and data were analysed using Flowjo (v. 7.6.5 and 10.6.1).

### Mosquito infections and genetic crosses

Stablilates with *P. berghei* 820 dilution cloned single ko lines (md1, md2, md3, md4, md5, gd1, fd1, fd2, fd3 and fd4) or wt parasites were revived and then infected blood was collected by cardiac puncture. For single parasite lines mice were directly infected by intra-peritoneal injection. For genetic crosses *md1, md3, md4, md5, gd1, fd1, fd2, fd3* and *fd4* knockout lines were mixed at a 1:1 ratio of iRBC with a *nek4* knockout^59^ or a *hap2* knockout^60^ prior to infection with the mixed parasite infected blood intraperitoneally. On day three post-infection (typically at 2-10% iRBC) parasitaemia was assessed by microscopic observations of Giemsa stained blood films and exflagellation evaluated as previously described.^60^ Infected mice were anesthetised and ~50 mosquitoes allowed feed on each mouse for 15-20 min at 19°C. Unfed mosquitoes were removed after 24 hours. Midguts were dissected and oocysts were counted using light microscopy on day nine to twelve days post-feeding. Backbites were performed only for those single ko feeds that produced oocysts

### Bulk-RNA-seq

The generation and initial analysis of the additional time points for the bulk RNA-seq dataset was performed as described previously.^6^ Briefly, the PB_GAMi_ line (engineered to overexpress the AP2-G transcription factor and undergo synchronous conversion into gametocytes upon the induction with rapamycin) was synchronised to the late schizont stage and either induced or treated with vehicle only. At different time points post induction, the blood containing the developing parasites was harvested. Plasmodipur filters (EuroProxima) were used to remove the leukocytes according to the manufacturer’s instructions and red blood cells were lysed by resuspension in ice-cold 13 E-lysis buffer (1.5M NH_4_Cl, 0.1M KHCO_3_, 0.01 EDTA). The resulting parasite pellet was washed with 1xPBS and stored in Trizol reagent for future RNA extraction. Independently, in order to generate the reference transcriptome of male and female gametocytes, the 820 reporter line,^20^ was used to sort 5x10^6^ male and female gametocytes, as described in the previous section. The resulting male and female pellets were also stored in Trizol for further processing. Complete RNA was isolated from all the samples using Trizol/chloroform extraction followed by isopropanol precipitation and 1-2 μg of starting material was taken for mRNA isolation and stranded RNA-seq library construction. The libraries were prepared using NEBNext library preparation modules, with minor modifications of the protocol shown to improve the yield when sequencing AT-rich transcriptome of *Plasmodium* parasites.^61^The samples were pooled and sequenced using an Illumina HiSeq 2500 system according to the manufacturer’s instructions. All samples were generated in biological duplicates or triplicates and uninduced controls were always generated and processed together with the induced samples.

### Smart-seq2 scRNA-seq

#### Cell preparation and staining

Knockout mutants for *md4, md5, gd1, fd2, fd3*, and *fd4* were created in a background constitutively expressing GFP. Each mutant was individually mixed in a 1:1 ratio (based on parasitemia counts) with mCherry_hsp70_ wild-type parasites constitutively expressing mCherry as internal control. Inclusion of a co-infecting wild-type strains allows to control forenvironmental influences on sex ratio, sexual commitment, as well as transcriptional variation between hosts. Mutants created in the *P. berghei* 820 background (*md1, md2, md3*, and *fd1*) cannot be combined with an internal mCherry control, but an external mCherry control in another mouse was included (Figure S2B). All mice were treated the same from this point onwards. 3 days after inoculation, mice were terminally bled by cardiac puncture. The ~1 mL blood sample was immediately transferred into a pre-warmed (37 °C) 1.5-mL tube, and transferred into a sealed culture flask containing 50 mL of schizont culture medium (RPMI with 20% FBS, 15 mM NaHCO_3_, and 1% penicillin/streptomycin). Parasites were cultured for 24 hours at 36.5 °C with shaking at 65 rpm. Cultures were harvested by centrifugation at 450 x *g* for 3 minutes at room temperature. Late-stage- and gametocyte- infected red blood cells were purified on a 55% Histodenz gradient by centrifugation at 300 × *g* for 20 minutes at room temperature. Cells were washed once and resuspended in 1 mL game-tocyte non-activation media.

#### Cell sorting

Sorting was performed as described in Reid et al.^62^ Briefly, 4 μL of lysis buffer (0.8% of RNAse-free Triton-X (Fisher) in nuclease-free water (Ambion)), UV-treated for 30 min with a Stratalinker UV Crosslinker 2400 at 200, 000 μJ/cm2, 2.5 mM dNTPs (Life Technologies), 2.5 μM of oligo(dT) (Non-anchored OligoDT, HPLC purified, 100 μM, 5’AAGCAGTGGTATCAACGCAGA GTACTTTTTTTTT TTTTTTTTTTTTTTTTTTTTT3’; IDT) and 2U of SuperRNAsin (Life Technologies)) was dispensed in 96-well plates. Cell sorting was performed on an Influx Cell Sorter (BD Biosciences) with a 70 μm nozzle. For mixed parasite populations,cells were sorted by gating for single-cell events, Hoechst positive events (compared to an uninfected RBC control), and then on GFP (mutant population) or mCherry (wild-type population). For 820-background parasites, this strategy was modified so that parasite cells were sorted by gating for single-cell events and on GFP (male), mCherry (female), or Hoechst (unbiased sort of all parasites of that genotype). A non-sorted negative control, and a positive 100-cell control were included on every plate.

#### Library preparation and sequencing

First and second strand cDNA synthesis and pre-amplification were performed as described in Reid et al.^62^ with 96-well plates and 25 PCR cycles. Quality control of cDNA samples was monitored using a high-sensitivity DNA chip on the Agilent 2100 Bioanalyzer. Libraries were prepared using dual indexes as described in Reid et al.^62^ and pools of 384 cells (4 plates) were sequenced.onto one lane on a HiSeq 4000 using v4 chemistry with 75 bases paired-end reads and run according to manufacturer’s instructions.

### 10x Genomics chromium scRNA-seq

mCherry_hsp70_ parasites were used for all experiments, so any data generated was comparable with Smart-seq2 data which used this background as a wild type control. Blood was obtained as previously described (3 days post infection), a leukodepletion step was included by using a pre-wetted Plasmodipur syringe filter (EuroProxima) was used for leukodepletion prior to culturing. To cover the whole span of sexual development and mitigate unequal representation due to sequestration, two separate cultures from two mice were set-up in a staggered way so that they could be harvested at the same time, respectively 30 minutes and 12 hours after blood harvest (Figure S2C).

Cultures were smeared prior to harvesting in order to ascertain precise parasitemia. After harvesting, the total number of red blood cells in each sample was counted using a single-use hemocytometer (NanoEntek). This count was corroborated using a Countess cell counter. The concentration of infected red blood cells (iRBCs), derived from the parasitaemias and red blood cell concentration,.in each culture was established and.cells were pooled 1:1. Cells were loaded according to the manufacturer’s instructions to recover 5000 cells. Chromium 10x v2 chemistry was used and the library was prepared according to manufacturer’s instructions and sequenced across 2 lanes of a HiSeq 2500 on Rapid Run settings using asymmetric paired-end sequencing (26 cycles for Read 1 and 98 cycles for Read 2).

### Bulk RNA-seq data analysis

The raw data processing, generation of initial *.cram files and adapter removal was performed using the default analysis pipelines of the Sanger Institute. The raw data was transformed into paired *. fastq files using Samtools software (v. 1.2).^63^ The generated reads were re-aligned to the *Plasmodium berghei* ANKA genome (PlasmoDB-30 release) in a splice-aware manner with HISAT2^46^ using the –known-splicesite-infile option within the splicing sites file generated based on the current genome annotation. Resulting *.bam files were sorted and indexed using Samtools (v. 1.2) and HT-seq python library (v. 1.3.1).^47^ was used to generate reads counts for all genes for further processing.

The matrix of gene counts was combined with the time points published previously.^6^ Differential expression analysis was performed at each time point between the induced and uninduced samples using R (v3.4.4) with DESeq2 package v 1.18.^48^ In parallel, the differential expression analysis was performed between male and female gametocytes as well as between each gametocyte sex and asexual parasites. In order to cluster the genes according to their responses to the AP2-G induction and sex specificity, the fold differences in gene expression at each time point as well as fold differences between male and female gametocytes were extracted from DEseq2 expression tables and used as input for the self-organising maps training algorithm implemented in the “kohonen” R package (ver. 2.0.14).^64^ The ‘som’ algorithm was run with 8x8 map size, 200 data presentation cycles and default “alpha” parameters. The relative expression of each of the 64 clusters was visualised using plotting functions implemented within the “kohonen” package. In order to confirm the separation of male and female expression clusters, genes classified as female-, male-, gametocyte- and asexual-specific, based on the differential expression were overlaid on the map in in order to visualise the clusters they belonged to. Genes were classified as male/female/asexual specific if they were differentially upregulated eg. females (with log2FC >2 and FDR<0.05) when compared to both asexual parasites and males. Genes overexpressed in gametocytes but without clear preference between the sexes were classified as gametocyte-specific. Full list of genes with their cluster assignments in Table S2.

### Mapping and generation of expression matrices for scRNA-seq data

#### Smart-seq2 mapping

Single-cell *Plasmodium* transcriptomes were mapped as reported previously.^62^ CRAM files were downloaded from iRODs Wellcome Sanger Institute core pipeline. CRAM files were converted to FASTQ format using Biobambam2 (v2.0.37)^65^ (*bamtofastq exclude= SECONDARY*,*SUPPLEMENTARY,QCFAIL*). Nextera adaptor sequences were trimmed using Trim Galore (v0.4.3)^66^ (*trim_galore -q 20 -a CTGTCTCTTATACACATCT –paired –stringency 3 –length 50 -e 0.1*). Trimmed FASTQ files were then mapped using HISAT2 (v2.1.0)^67^ and indexes were produced using the *P. berghei* v3 genome sequences,^68^ downloaded from GeneDB^69^ (October 2016). Trimmed reads were then mapped using default parameters (*hisat2 –max-intronlen 5000 -p 12 -q -x*). GFF files were downloaded from GeneDB (October 2016) and converted to GTF files using an in-house script. All feature types (mRNA, rRNA, tRNA, snRNA, SnoRNA, pseudogenic_transcript and ncRNA) were conserved, with their individual ‘coding’ regions labelled as CDS in every case for convenience. Where multiple transcripts were annotated for an individual gene, only the primary transcript was considered. Reads were summed against genes using HTSeq (v0.11.2)^47^ (*htseq-count -f bam -r pos -s no -t CDS*). HTSeq excludes multimapping reads by default (-a 10). This means that reads mapping ambiguously to similar genes from the same family are not considered in our analysis.

#### 10x data alignment, cell barcode assignment, and UMI counting

The sequencing reads in CRAM format were downloaded from iRODs Wellcome Sanger Institute core pipeline. CRAM files were converted to FASTQ format using the samtools fastq command.^63^ Cell Ranger (version 2.1.1) was used to create a reference file from the *P. berghei* v3 genome (obtained from: www.sanger.ac.uk/resources/downloads/protozoa/).^68^ using standard parameters (*cellranger mkref –genome=*.. *–fasta=*.. *–genes=*..). The gene PBANKA_0713500 was manually corrected to include two exons with missing ids. FASTQ files were passed into the Cell Ranger 2.1.1 workflow to assign each read to a cell barcode and UMI using standard parameters ^70^ (*cellranger count –id=*.. *–transcriptome=*.. *-fastq=*..).

### Filtering and normalization of scRNA-seq data

R version 4.0.3 (2020-10-10) was used for all scRNA-seq analysis.^71^

#### Smart-seq2 filtering

Count matrices and associated metadata (phenodata) were read into R (version 4.0.3) and no cell and 100 cell controls were removed from further analysis. Seurat (version 3.2.2) was used for pre-processing.^49^ 63/5245 genes were not detected in any cell and were also removed. Cells with genes per cell < 220, genes per cell > 3300, percentage of total counts mapping to mitochondrial genes > 20%, and number of counts per cell < 1000 were removed. This resulted in the removal of 706/3450 cells.

#### 10x filtering

The raw output barcodes, genes and matrix files were read into Seurat (v3.2.2) using the Read10X command.^49^ To distinguish cells from background, an expectation–maximization (EM) algorithm was applied using mixtools (v.1.2.0)^50^ to discover where the two distributions (cells and background) intersected. This resulted in the identification of 7762 cells. Low-quality cells were then filtered by removing any cell that contained <200 genes per cell; this removed 1131 cells resulting in 6631 cells.

#### Normalization and doublet detection

In both datasets cells were normalized (*NormalizeData(x, normalization.method = “LogNormalize”, scale.factor = 10000*), variable genes were found (*FindVariableFeatures(x, selection.method = “vst”, nfeatures = 2000*), and data was scaled (all.genes = all genes in the datset; i.e. *ScaleData(x, features = all.genes)*). For the 10x data, doublets (cell barcodes that are associated with a significant number of reads from multiple cells) were filtered out using DoubletFinder.v3^72^ (*doubletFinder_v3(pb_sex, PCs = 1:21, pN = 0.25, pK = 0.01, nExp = nExp_poi, reuse.pANN = FALSE, sct = FALSE*). Doublet removal was not applied to the Smart-seq2 dataset as a singlet gate was applied during FACS.

### Single-cell transcriptome analysis of wild-type data

#### Data integration

Wild-type cells from the Smart-seq2 and 10x datasets were integrated using Seurat v3.2.2.^49^ Each dataset was subsetted to only include genes that were present in both (5018 shared genes). Datasets were then individually normalised (*NormalizeData*()) and variable features were found (*FindVariableFeatures(x, selection.method = “vst”, nfeatures = 2000*). Anchors were found (*FindIntegratio-nAnchors(object.list = x, dims = 1:21*) and integration was performed (*IntegrateData(anchorset = x, dims = 1:21, features.to.integrate = shared.genes; where shared.genes is all 5018 genes*).

#### Cell projection, clustering, and annotation

To identify subpopulations of cells in the integrated dataset, PCA was performed (RunPCA(x, npcs = 30) and then cells were projected into two dimensions using the UMAP algorithm^54^ using the first 10 principal components after inspection of an elbow plot to detect significant principal components in Seurat v3.2.2; the other parameters used were: n.neighbors = 150, min.dist = 0.4, repulsion.strength = 0.03, local.connectivity = 150. Cells were clustered in a ten dimension UMAP space using the Louvain algorithm with multilevel refinement at a resolution of 2.^73^

Cells were manually annotated by assigning each Louvain cluster to either asexual, progenitor, male or female, based on established marker genes (Figure S3). All wild-type cells were then ordered along pseudotime using Monocle 3 (version 0.2.3.0)^51–53^ (learn_graph(x, learn_graph_control=list(ncenter=550, minimal_branch_len = 15), use_partition = FALSE; order_cells(x)). The root cells were selected manually using the interactive feature. The median pseudotime value for each cluster was then used to order clusters within each lineage. At this point, two female clusters collapsed into one due to their similar median pseudotime.

The progenitor cluster was analysed further (n = 206, all cells were from the 10x dataset). Variable features were calculated for this subset and the cells were projected into 30-dimension PCA space. The elbow plot of these PCs was inspected and seven of these dimensions were used to find neighbours. Clusters were generated using the Louvain algorithm with multilevel refinement at a resolution of 0.5.(75) (Figure S4A.) Marker genes for these resulting two clusters were found using the MAST framework implemented in Seurat v.3.2.2 (FindAllMarkers(x, only.pos = FALSE, min.pct = 0.25, logfc.threshold = 0.25, test.use = “MAST”)^74^

#### Pseudotime and gene module generation

In order to analyse the sexual branch, the following clusters were subsetted for further analysis: Asexual_10, Asexual_11, Asexual_12, Progenitor, Male_1, Male_2, Female_1, Female_2, Female_3. 9 outlier cells were removed from analysis as they were located far from the clusters and would have affected pseudotime calculation. This resulted in a subset of 2817 branch cells (Figure 3C).

For the pseudotime analysis of the sex branch, the subsetted cells were further subsetted so that only wild-type cells generated using 10x were included in the analysis. The reason 10x cells were only used in following steps was because there is not a robust way to account for the batch effects between the Smart-seq2 and 10x dataset within Monocle and Monocle is not compatible with negative values generated during Seurat batch correction. The subsetted object counts matrix was preprocessed (preprocess_cds(x, num_dim = 50, norm_method = “log”)) and UMAP coordinates were extracted from the Seurat object. The graph structure was learnt (learn_graph(x, l learn_graph_control=list(ncenter=550, minimal_branch_len = 30), use_partition = FALSE)). The cells were ordered along pseudotime (order_cells(x)) and the root cells were selected manually using the interactive feature. The 23 gene modules were generated by first performing graph_test(x, neighbor_graph=“principal_graph”, cores=8, expression_family = “negbinomial”). Significant genes were selected by removing any genes with a *q*-value > 0.05. Gene modules were then found using find_gene_modules(x[significant_genes,]], resolution=c(10^^^seq(-6,2)), random_seed = 1234), to maximise the number of modules as suggested by the developers of Monocle 3.

#### Enrichment of gene classes in gene modules

A list of the gametocyte screen hits was used to find the percentage of hits in each module, which was given by the number of screen hits present in that module divided by the number of genes that were screened in that module. The percentage of DOZI-regulated genes per module was calculated by extracting the genes identified in both of the DOZI and CITH fractions of RIP-Chip experiments performed in,^25^ and calculating the percentage of these genes that appear in each module. The percentage of genes per module for each of the asexual phenotypes identified in.^16^was also calculated for genes that were covered in that screen. The significance of the module based on the DOZI/CITH-associated genes, gametocyte screen hits, and asexual screen hits, respectively, was calculated using the hypergeometric probability density function (P), as shown above in the Bulk RNA-seq data analysis section. In this case, *k* and K are the numbers of gene hits and the number of total genes in a given module, respectively, and n and N are the total number of hits in the screen and the total number of genes in screen, respectively. For each module the *p*-value was calculated by sum over probabilities for greater or equal than the enriched number of sex-specific genes to test the null hypothesis (equation 2)…

### Single-cell transcriptome analysis of single knockout mutant data

#### Data integration

To compare mutant transcriptomes to wild-type ones, all cells from the Smart-seq2 and 10x datasets were integrated using Seurat v3.2.20.^49^ Each dataset was subsetted to only include genes that were present in both (5018 shared genes). Datasets were then individually normalised (NormalizeData()) and variable features were found (FindVariableFeatures(x, selection.method = “vst”, nfeatures = 2000). Anchors were found (FindIntegrationAnchors(object.list = x, dims = 1:21) and integration was performed (IntegrateData(anchorset = x, dims = 1:21, features.to.integrate = shared.genes; where shared.genes is all 5018 genes).

#### Cell projection, clustering, and sex branch isolation

To identify subpopulations of cells in the integrated dataset, PCA was performed (RunPCA(x, npcs = 30) and then cells were projected into two dimensions using the UMAP algorithm^54^ using the first 10 principal components after inspection of an elbow plot to detect significant principal components in Seurat v3.20.20; the other parameters used were: n.neighbors = 150, min.dist = 0.4, repulsion.strength = 0.03, local.connectivity = 150. Cells were clustered in a ten dimension UMAP space using the Louvain algorithm with multilevel refinement at a resolution of 0.5 (75) (Figure S5A).

In order to analyse the sexual branch, 11/26 clusters were subsetted for further analysis (Figure S5D). 8 outlier cells were removed from analysis as they were located far from the clusters and would have affected pseudotime calculation later. The identities of these 8 cells were checked to ensure that they were not enriched in a specific mutant and they contained a mixture of wild-type and mutant cells. This resulted in a subset of 3012 sex branch cells. The cells were projected into 2 dimensional PCA space and 17 new clusters were generated using the first 11 principal components, and the Louvain algorithm with multilevel refinement at a resolution of 1. Each cluster was then assigned a sex identity based on the position in the PCA plot and the detection of known marker genes. Pseudotime was then calculated using Monocle 3 (version 0.2.3.0) (79–81) by first learning the graph structure (learn_graph(x, learn_graph_control = list(ncenter = 200, minimal_branch_len = 10), use_partition = FALSE)). The cells were ordered along pseudotime (order_cells(x)) and the root cells were selected manually using the interactive feature. Cells were assigned to one of the three resultant branches using choose_graph_segments() in Monocle 3. Finally, cells were assigned an identity if they belonged to both a cluster that was designated as male, female or progenitor as detailed above and also belonged to the male, female or progenitor branch, respectively.

#### Differential gene expression analysis

In order to assess how mutants impacted gene expression, differential expression analysis was performed using Seurat v3.2.2 and MAST.^74^ In order to compare cells from similar points of development, the latest cluster in development in the sexual branch that the mutant affected with representation of the mutant under comparison was chosen. Within this cluster, the mutant cells were directly compared to the wild-type cells (Figure S6). For *fd2*, both clusters 15 and 8 were chosen because the representation of wild-type cells in cluster 15 was too low.

### Immunofluorescence microscopy

Rabbit antibodies to the DDX6 RNA helicase (DOZI) PBANKA_1217700 were raised (Proteintech) against the highly conserved peptide LAGKNILARAKNGTGKTAA representing positions 95-113 of the mature protein. Antibodies were affinity-purified from serum using protein A sepharose and used at a 1:500 dilution.

*Plasmodium berghei* parasites were smeared on a glass slide and allowed to air-dry, fixed in 4% paraformaldehyde (prepared in 1x PBS), before washing in 1x PBS. Samples were permabilized in 0.2% Triton-X in PBS for 5 mins, before blocking in 5% (w/v) FBS + 2% (w/v) milk in PBS for 30 mins. Rabbit anti-DOZI and chicken anti-GFP antibodies were applied diluted 1:500 in blocking solution (as above) and incubated overnight at 4 °C in a humid chamber. Following further washes in PBS, secondary antibodies (goat antirabbit Alexafluor 594, goat anti-chicken Alexafluor 488) were applied at 1:2000 dilution in blocking solution, and incubated at room temperature for 2 hours in the dark. Slides were washed further in 1x PBS before mounting coverslips with Vectashield + DAPI (Vector labs, Cat. No. H-1000). Samples were visualized on a the DeltaVision Core wide-field fluorescence microscope at 100x magnification, and z-stack images deconvolved and projected to 2D with SoftXoRx software (Applied Precision). Parasites were fixed in paraformaldehyde and nuclei are stained using DAPI.

### Immunoprecipitation and mass spectrometry analysis

Peptides were analyzed with online nanoLC-MS/MS on an Orbitrap Fusion (MH01) or Lumos (MH02) Tribrid mass spectrometer coupled with an Ultimate 3000 RSLCnano System. Samples were first loaded and desalted on a nanotrap (100 μm id x 2 cm) (PepMap C18, 5 μm, 100A) at 10 μl/min with 0.1% formic acid for 10 min and then separated on an analytical column (75 μm id x 50 cm) (PepMap C18, 5 μm, 100A) over a 90 min linear gradient of 4-32% CH3CN/0.1% formic acid at 300 nL/min, and the total cycle time was 110 min. The Orbitrap Fusion and Lumos were operated in standard data-dependent acquisition. Precursors between 375 and 1,500 m/z were selected, with mass resolution of 120,000, automatic gain control of 4 3 105, and IT (injection time) of 50 ms, with the top speed mode in 3 s, and the precursors were fragmented in HCD (higher collision dissociation) cell at 32% collision energy with a quadrupole isolation width of 1.6 Th (Thomson unit). Targeted precursors were dynamically excluded for further isolation and activation for 40 s with 10 ppm mass tolerance.

Raw files were processed with Proteome Discoverer v. 2.4 (Thermo Fisher). Database searches were performed with Sequest HT against the PlasmoDB-54 Plasmodium berghei ANKA annotated proteins (v. October 2021) appended with the cRAP database (www.thegpm.org/crap/). The search parameters were set to trypsin digestion, 2 missed cleavages, 10 ppm mass tolerance for MS, 0.5 Da mass tolerance for MS/MS, with variable modifications of protein N-terminal acetylation, oxidation(M), and pyro-glu (N-term Q). Peptide false discovery rates (FDR) were estimated based on matches to reversed sequences in a concatenated target-decoy database using Percolator and set at 0.01. Protein identification required at least one high-confidence peptide at FDR <1%. Protein hits from the cRAP database were removed from the lists prior to further analysis. To discriminate bait-specific interactions from background binding, protein lists from bait and control experiments were analysed with SAINTexpress.^55^ Proteins with a SAINT probability score (SP) ≥ 0.99 were deemed high confidence interacting proteins. The mass spectrometry proteomics data have been deposited to the ProteomeXchange Consortium via the PRIDE^75^ partner repository with the dataset identifier PXD033827.

### Fractionation of *Plasmodium* proteins into cytoplasmic, nuclear and nuclear insoluble extracts

Fractionation of cytosolic and nuclear proteins was done as follows and largely followed the published procedure for *P. falciparum*.^28^ Parasites were isolated from red blood cells using ice-cold erythrocyte lysis buffer (150 mM NH_4_Cl, 10 mM KHCO_3_, 1 mM EDTA) and subsequently washed once with ice-cold 1x PBS. To isolate cytosolic proteins, washed parasite pellets were resuspended with 5x the pellet volume (PV) of ice-cold lysis buffer (20 mM HEPES (pH 7.8), 10 mM KCl, 1 mM EDTA, 0.65% (v/v) IGEPAL CA-630, 1 mM DTT, 1x cOmplete Protease Inhibitor Cocktail) and incubated for 5 min on ice. Cell lysates were spun for 10 min at 2,500 x g (4°C), the supernatant (i.e. cytosolic fraction) was transferred into a new tube and stored at -20°C. The nuclear pellet was washed twice with 5 PVs lysis buffer and subsequently resuspended in 5 PV ice-cold extraction buffer (50 mM Tris-HCl (pH 7.8), 150 mM NaCl, 3 mM MgCl2, 1.0% (v/v) NP-40, 1x cOmplete Protease Inhibitor Cocktail). Protein samples were sonicated using a Bioruptor Sonicator (Diagenode) for 5 cycles of 30s ON and 60s OFF (‘High-Power’ setting). Following sonication, 5 U/μl Benzonase (Merck Millipore) were added and lysates were incubated for 30 min on ice with occasional vigorous mixing. Subsequently, the NaCl concentration was adjusted to 600 mM using a 5 M NaCl stock solution and the lysates were incubated for another 30 min on ice with occasional vigorous mixing. To isolate the soluble nuclear proteins, lysates were centrifuged for 20 min at 20,000 x g (4°C), the supernatant (i.e. soluble nuclear fraction) was transferred to a new tube and stored at -20°C. To isolate the insoluble nuclear protein fraction, the insoluble pellet was resuspended in 5 PV of 2x SDS loading buffer (100 mM Tris-HCl (pH 6.8), 12% (v/v) glycerol, 0.008% (w/v) bromophenol blue, 2% (w/v) SDS, 2% (v/v) β -mercaptoethanol) and heated for 10 min to 99°C. The mixture was then briefly chilled on ice, spun for 20 min at 20,000 x g (4°C) and the supernatant (i.e. insoluble nuclear fraction) was transferred into a new tube and stored at -20°C. Total cellular proteins were isolated from parasites using RIPA buffer lysis. Parasite pellets were resuspended in 5 PV ice-cold RIPA buffer (50 mM Tris–HCl (pH 8.0), 150 mM NaCl, 1 mM EDTA, 0.5% (w/v) sodium deoxycholate, 0.1% (w/v) SDS, 1% (v/v) triton X-100, 1 mM DTT, 1x cOmplete Protease Inhibitor Cocktail) and incubated for 30 min on ice. Lysates were centrifuged for 20 min at 20,000 x g (4°C), total protein containing supernatant was transferred into a new tube and stored at -20°C.

## Quantification And Statistical Analysis

The significance of the cluster based on the sex-specific genes was calculated using the hypergeometric probability density function (P):

(Equation 1) $$P(\text{k},\text{N},\text{K},\text{n})=\frac{\left(\begin{array}{c} K\\ k \end{array})(\begin{array}{c} N-K\\ n-k \end{array}\right)}{\left(\begin{array}{c} N\\ n \end{array}\right)}$$

(Equation 2) $$pvalue=\overset{k}{\underset{K}{\sum }}P(x)$$

where *k* and K are the numbers of sex-specific genes and the number of total genes in a given cluster, respectively, and n and N are the total number of sex-specific genes in the screen and the total number of genes in screen, respectively. For each cluster the p-value was calculated by sum over probabilities for greater or equal than the enriched number of sex-specific genes to test the null hypothesis (Equation 2). Methods used for computing statistical significance and the representation of n are indicated in figure legends. Statistical significance was considered for p values below 0.05. Data was analyzed using GraphPad Prism Software and R.

## Additional Resources

Screen data can be and visualized on the *Plasmo*GEM website https://plasmogem.shinyapps.io/Gametocytes_Shiny/. Single-cell RNA-seq data can be searched and visualised on the Malaria Cell Atlas website www.malariacellatlas.org. Mutant scRNA-seq data can be interacted with on the cellxgene instance http://obilab.molbiol.umu.se/gcsko.

## Supplementary Material

Table S1

Table S2

Table S3

Table S4

Table S5

Table S6

Supplemental information

## Data Availability

The raw scRNA-seq data for this study have been deposited under accession number European Nucleotide Archive: PRJEB44892 https://www.ebi.ac.uk/ena/browser/view/PRJEB44892 and raw bulk RNA-seq data are available under accession numbers Gene Expression Omnibus: GSE110201, GSE168817. Accession numbers are also listed in the key resources table. Microscopy data reported in this paper will be shared by the lead contact upon request. Mass spectrometry proteomics data have been deposited to the ProteomeXchange Consortium via the PRIDE partner repository with the dataset identifier PRoteomics IDEntifications database: PXD033827. Supporting files and code are available on Github at https://github.com/andyrussell/Gametocytogenesis and is publicly available as of the date of publication. DOIs are listed in the key resources table. Any additional information required to reanalyze the data reported in this paper is available from the lead contact upon request.

## Highlights

- A genome-scale screen identifies genes for *Plasmodium berghei* sexual development
- scRNA-seq phenotyping places mutants in the context of a transcriptomic atlas
- A LOTUS/OST-HTH domain gene is a conserved factor determining male sex
- Protein interactors point to posttranscriptional mechanisms for sex determination
