# Supplementary material for: Regulators of male and female sexual development are critical for the transmission of a malaria parasite
Source: Cell Host Microbe. Author manuscript; Available in PMC 2024 Jun 12. (PMC7616090; doi:10.1016/j.chom.2022.12.011)
Supplement: Supplemental information [file EMS196587-supplement-Supplemental_information.pdf]

Cell Host & Microbe, Volume *31*

Supplemental information

Regulators of male and female sexual development are critical for the transmission of a malaria parasite

Andrew J.C. Russell, Theo Sanderson, Ellen Bushell, Arthur M. Talman, Burcu Anar, Gareth Girling, Mirjam Hunziker, Robyn S. Kent, Julie S. Martin, Tom Metcalf, Ruddy Montandon, Vikash Pandey, Mercedes Pardo, A. Brett Roberts, Claire Sayers, Frank Schwach, Jyoti S. Choudhary, Julian C. Rayner, Thierry Voet, Katarzyna K. Modrzynska, Andrew P. Waters, Mara K.N. Lawniczak, and Oliver Billker

Supplementary Figures

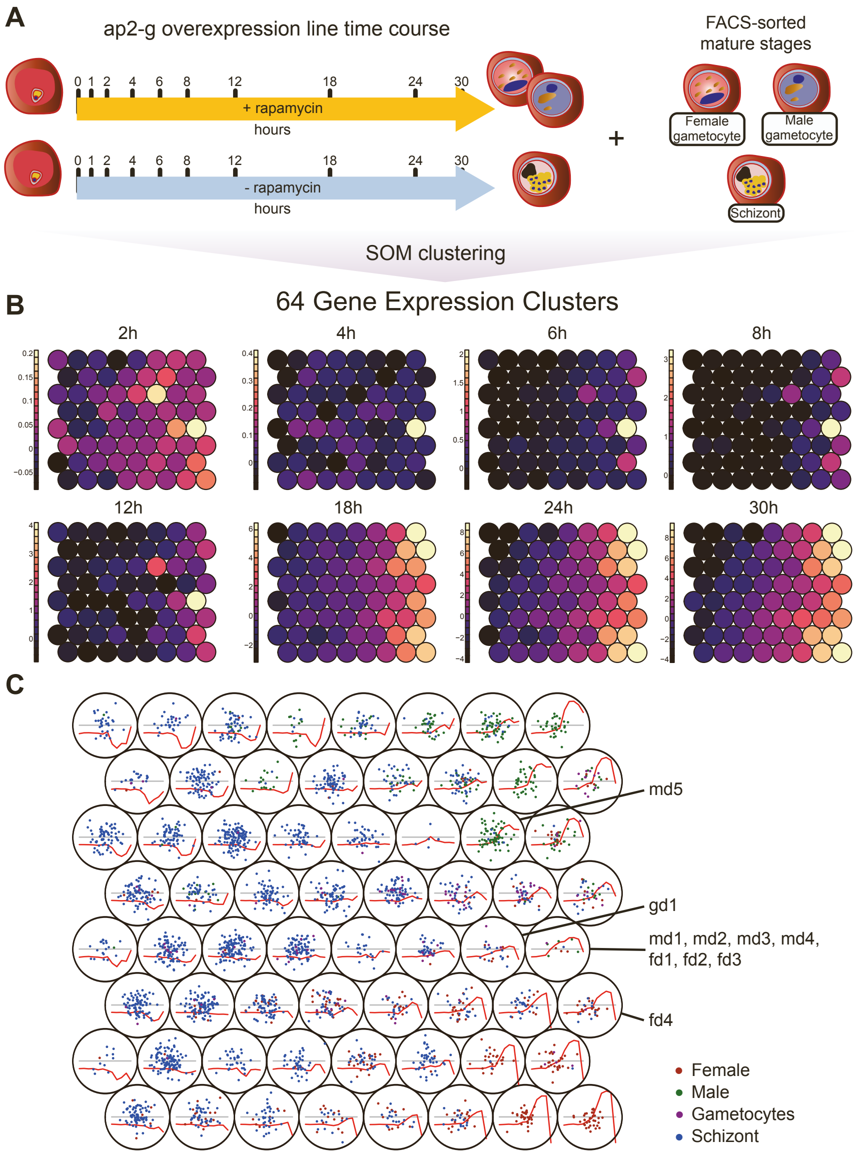

D

	0h	1h	2h	4h	6h	8h	12h	18h	24h	30h	44h
Cluster 1	1	2	2	0	0	0	0	0	0	0	0
Cluster 2	1	2	0	0	0	0	0	0	0	0	0
Cluster 3	2	0	0	0	0	0	0	0	0	0	0
Cluster 4	0	2	0	0	0	0	0	0	0	0	0
Cluster 5	0	3	0	2	0	0	0	0	0	0	0
Cluster 6	0	2	0	0	0	0	0	0	0	0	0
Cluster 7	0	0	2	2	0	0	2	0	0	3	10
Cluster 8	2	0	2	0	0	0	17	6	44	50	70
Cluster 9	7	0	0	0	0	0	0	0	0	0	0
Cluster 10	0	0	1	1	0	0	0	0	0	0	0
Cluster 11	0	0	0	0	0	0	0	0	0	0	0
Cluster 12	0	0	2	0	0	0	0	0	0	0	0
Cluster 13	3	1	1	1	0	0	0	0	0	0	0
Cluster 14	0	0	2	0	0	2	0	0	0	0	0
Cluster 15	0	0	4	2	0	0	0	0	0	0	0
Cluster 16	0	0	9	5	18	21	21	5	2	2	2
Cluster 17	2	0	3	0	0	0	0	0	0	0	0
Cluster 18	0	1	0	0	0	0	0	0	0	0	0
Cluster 19	0	0	2	1	0	0	0	0	0	0	0
Cluster 20	1	0	0	1	0	0	0	0	0	0	0
Cluster 21	0	0	2	0	0	0	0	0	0	0	0
Cluster 22	2	0	3	0	3	0	2	0	0	0	0
Cluster 23	2	0	2	0	0	0	4	0	0	0	0
Cluster 24	2	0	2	6	13	17	4	2	0	0	0
Cluster 25	0	0	7	0	0	0	0	0	0	0	0
Cluster 26	3	9	0	0	0	0	0	0	0	0	0
Cluster 27	1	5	0	0	0	0	0	0	0	0	0
Cluster 28	2	1	0	0	0	0	0	0	0	0	0
Cluster 29	4	0	0	0	0	0	0	0	0	0	0
Cluster 30	0	0	4	1	0	0	0	0	0	0	0
Cluster 31	0	0	6	18	18	6	6	0	0	0	0
Cluster 32	0	0	42	59	84	101	93	0	0	0	0
Cluster 33	0	1	0	0	0	0	0	0	0	0	0
Cluster 34	1	2	1	2	0	0	0	0	0	0	0
Cluster 35	2	0	1	0	0	0	0	0	0	0	0
Cluster 36	0	1	0	0	0	0	0	0	0	0	0
Cluster 37	1	0	0	1	0	0	0	0	0	0	0
Cluster 38	0	0	0	0	0	0	0	0	0	0	0
Cluster 39	1	0	0	0	0	0	0	0	0	0	0
Cluster 40	4	0	2	8	2	0	2	0	0	0	0
Cluster 41	1	1	0	0	0	0	0	0	0	0	0
Cluster 42	1	0	0	0	0	0	0	0	0	0	0
Cluster 43	1	1	0	0	0	0	0	0	0	0	0
Cluster 44	2	0	0	0	0	0	0	0	0	0	0
Cluster 45	0	0	1	0	0	0	0	0	0	0	0
Cluster 46	0	0	27	20	13	13	27	0	0	0	0
Cluster 47	4	0	1	9	4	3	1	0	0	0	0
Cluster 48	3	0	0	0	3	5	0	3	5	3	0
Cluster 49	0	0	0	0	0	0	0	0	0	0	0
Cluster 50	0	1	0	1	0	0	0	0	0	0	0
Cluster 51	0	0	0	0	0	0	0	0	0	0	0
Cluster 52	0	1	0	0	0	0	0	0	0	0	0
Cluster 53	1	1	0	1	0	0	0	0	0	0	0
Cluster 54	0	0	0	1	0	0	0	0	0	0	0
Cluster 55	0	0	3	9	3	3	0	9	0	0	0
Cluster 56	0	0	0	0	26	26	15	67	34	34	11
Cluster 57	0	0	0	0	0	0	0	0	0	0	0
Cluster 58	0	0	0	0	0	0	0	0	0	0	0
Cluster 59	3	2	0	0	0	0	0	0	0	0	0
Cluster 60	4	0	0	0	0	0	0	0	0	0	0
Cluster 61	2	0	0	0	0	0	0	0	0	0	0
Cluster 62	0	0	0	0	0	0	0	0	0	0	0
Cluster 63	2	0	3	0	2	0	0	0	0	0	0
Cluster 64	0	0	6	6	6	13	9	70	44	32	6

Figure S1. Time course of bulk transcriptome changes during induced gametocytogenesis reveals clusters of genes co-regulated during early gametocyte differentiation. Related to Figure 2. (A) Outline of the experimental design showing samples collected from the induced (+ rapamycin) and uninduced (-rapamycin) ap2-g overexpression line [S1] as well as purified mature stages controls (schizonts and male/female gametocytes) (**B, C**) Maps generated using the self-organizing map (SOM) algorithm, showing 64 clusters of genes co-regulated during male and female gametocyte development. The relative expression of each of the clusters in the induced/uninduced population is represented by colors (**B**) or red lines (**C**). Points in (**C**) represent genes classified based on the differential expression between the mature stages as upregulated in schizonts (green), male/female (blue/red) gametocytes or gametocytes of both sexes (purple). Cluster assignment of key sex determinants discussed in the manuscript are marked in (**C**) Cluster assignment and expression levels of individual genes in the Table S2. Lines show the mean relative expression values of the cluster over time. (**D**) Heatmap showing fold enrichment of each cluster among the top 50 upregulated genes at each time point.

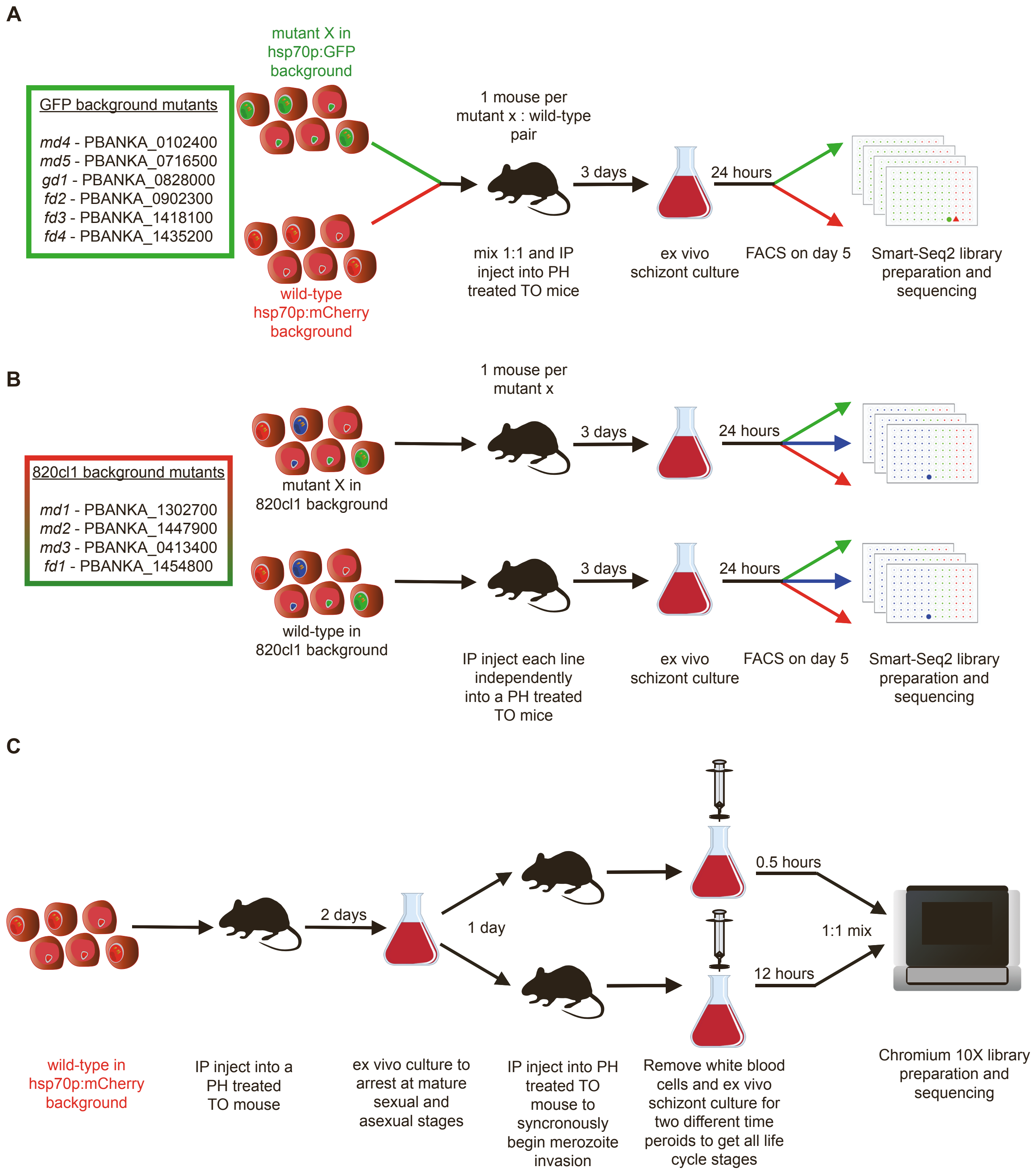

Figure S2. Design of scRNA-seq experiments. Related to Figure 3 and 4 (A, B) Smart-seq2 experiments use *in vitro* culture to allow parasites to differentiate along their determined trajectory without risking splenic clearance of developmentally aberrant mutants. Two different backgrounds were used in these experiments. **(C)** 10x experiments were set up to capture cells from intermediate stages of gametocyte differentiation.

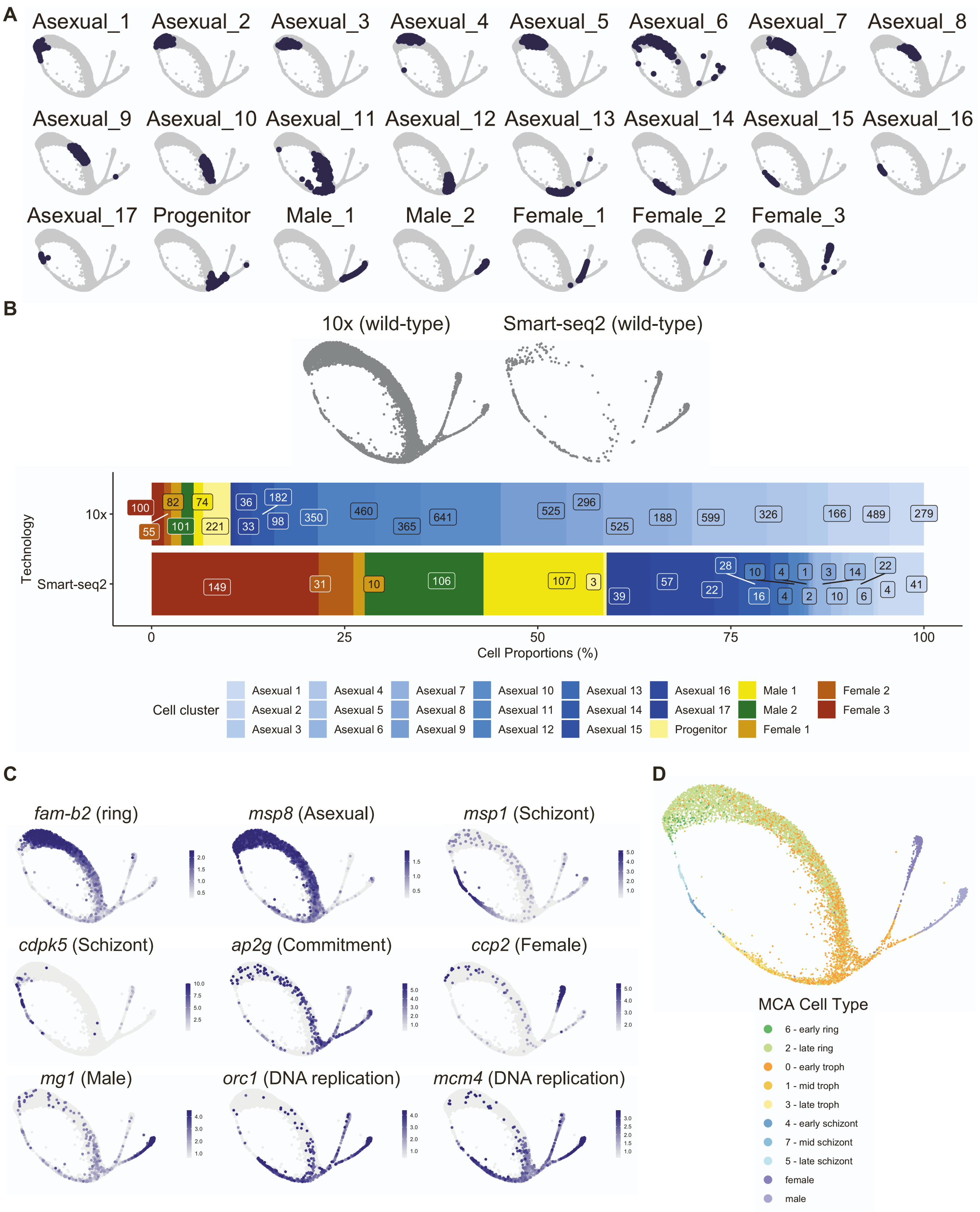

Figure S3. Wild-type scRNA-seq Data. Related to Figure 3. (A) The position of cells on the UMAP for each cluster is shown. **(B)** (top) Deconstructed UMAPs showing contribution of 10x and Smart-seq2 data to the combined analysis. (bottom) bar plot showing the proportion of each cell cluster captured using each technology. Together, these show how Smart-seq2 data captures predominantly later stage parasites, whereas 10x captures all stages. **(C)** The expression of known marker genes in wild-type cells is shown with the 5th and 95th quantiles set as the minimum and maximum expression values respectively to eliminate any outliers having a strong influence on visualisation. Examples include: Fam-b2, msp8, msp1, CDPK5, ap2-g, ccp2, mg1, orc1, mcm4. **(D)** Cell types defined by the malaria cell atlas [S2] were used as a reference to map our complete blood-stage

atlas. The original Malaria Cell Atlas did not contain developing gametocytes which is why these cells in the dataset associate most closely to trophozoites.

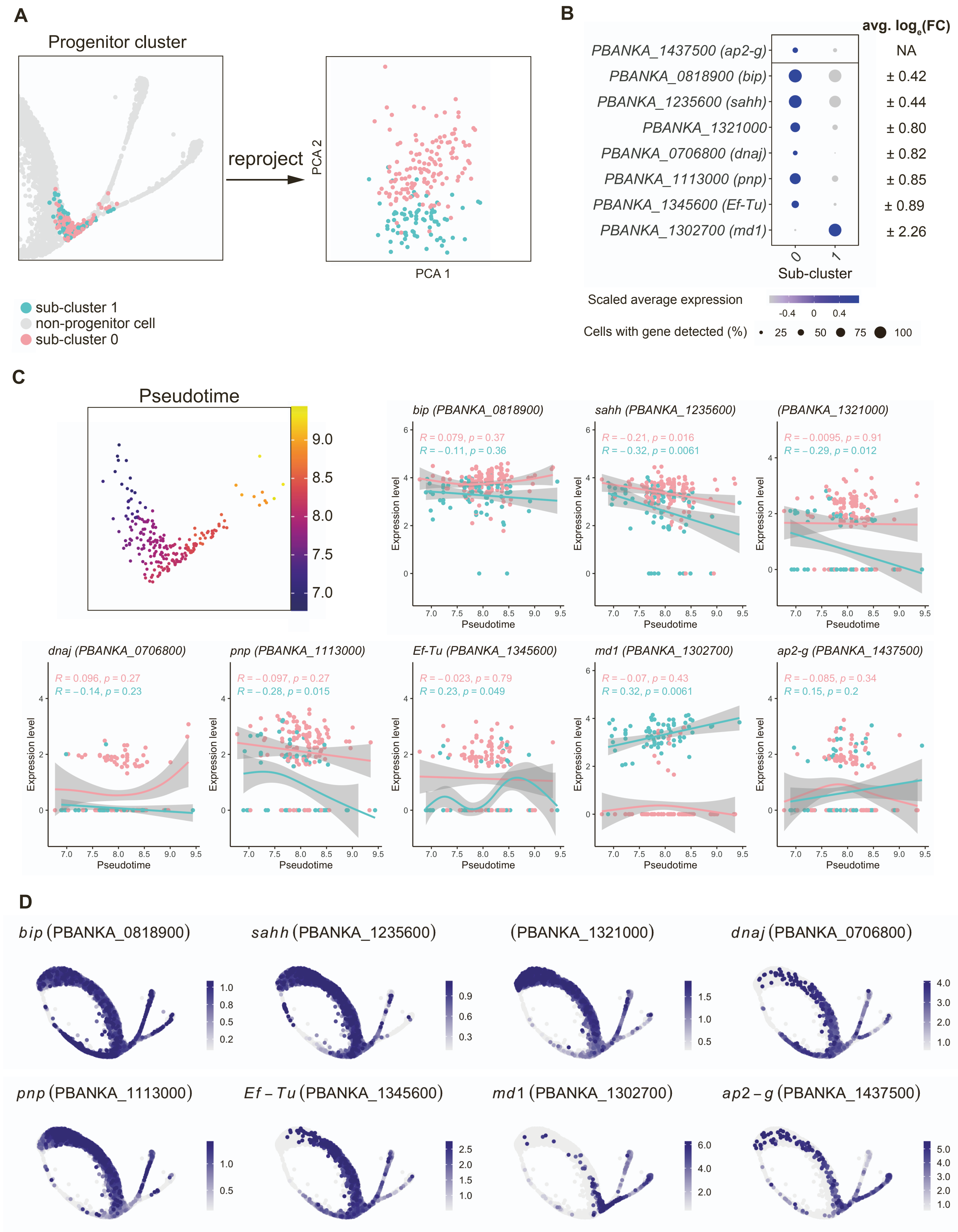

Figure S4. Examination of the bipotential branch. Related to Figure 3. (A) The progenitor cluster was subsetted and re-projected into PCA space. The subset was re-clustered to look for subpopulations (i.e. cells that have committed to one sex over the other) and two subclusters were generated, 0 and 1. **(B)** Marker genes

were calculated for each cluster and the expression of statistically-significant (adjusted $p < 0.05$, using Model-based Analysis of Single-cell Transcriptomics (MAST) [S3] with bonferroni correction) genes is shown in the dot plot. The only gene with an average $\log_2$(fold-change) > ±1 was *md1*. *ap2-g* is shown for comparative purposes but was not significantly differentially expressed between the two sub-clusters. **(C)** The cells are undergoing a developmental process in the bipotential cluster (left). To ensure that the two sub-clusters did not arise through two phases in progenitor progression over pseudotime, the expression of *md1* and *ap2-g* is plotted against pseudotime (right). Although the expression of *md-1* does increase over pseudotime in sub-cluster 1, the cells of the two subpopulations are dispersed throughout pseudotime. **(D)** To investigate whether the marker genes may have sex-specific roles, their expression is plotted All sub-cluster 0 positive markers show expression in the female branch, with some displaying stark contrast between branches (e.g. *sahh*).

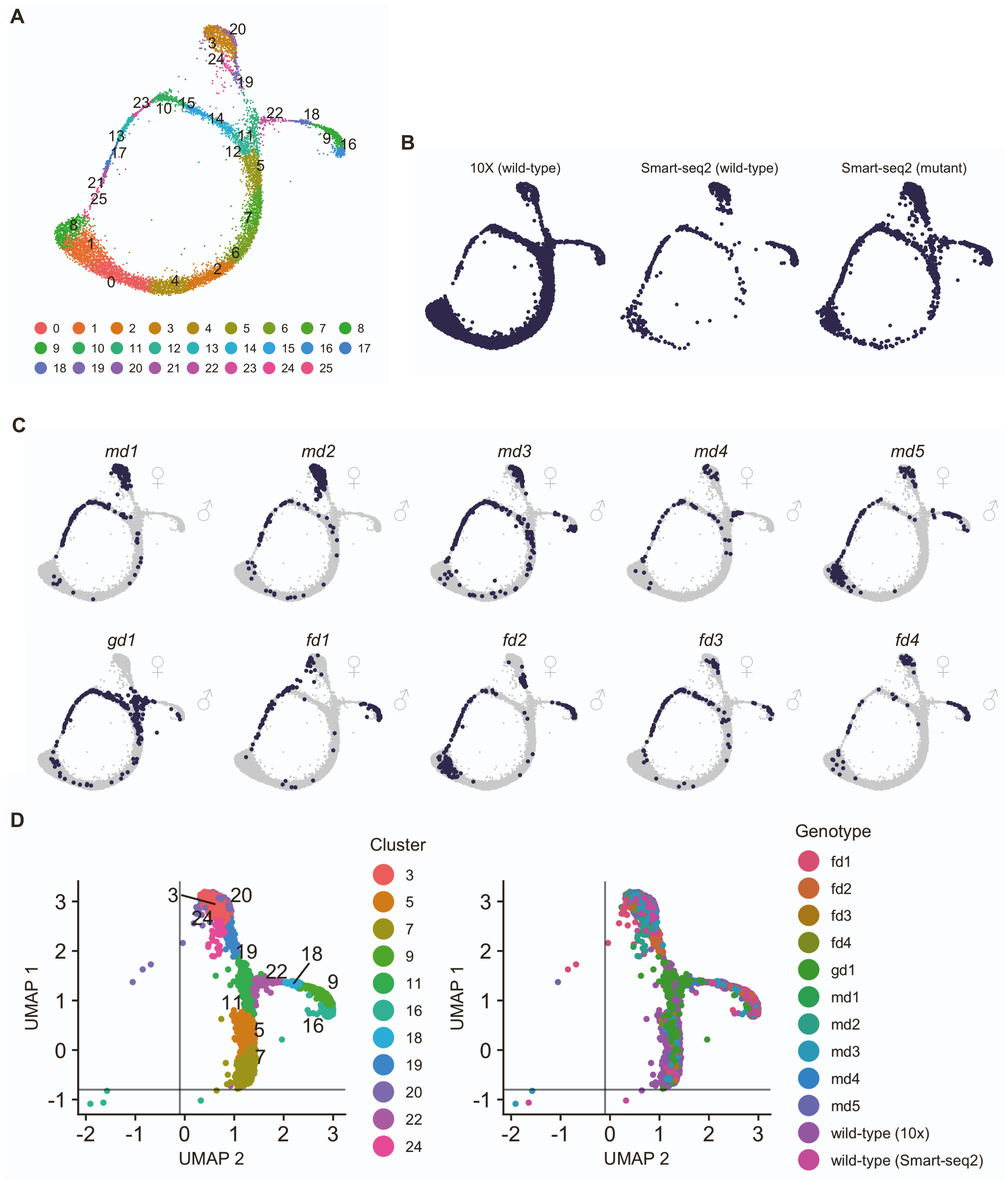

Figure S5. Integration of Smart-seq2 and 10x scRNA-seq Data. Related to Figure 4. (A) UMAP plot of all 8908 cells combined and coloured according to their cluster. **(B)** Deconstructed UMAPs showing contribution of 10x and Smart-seq2 data to the combined analysis. **(C)** UMAP plots highlighting the position of cells belonging to the mutant genotype denoted at the top of the plot. **(D)** UMAP plots showing the cells selected for the sexual branch analysis. Lines show the cutoffs used to remove the 8 outlier cells. Each plot is coloured by cluster membership (left) or genotype (right).

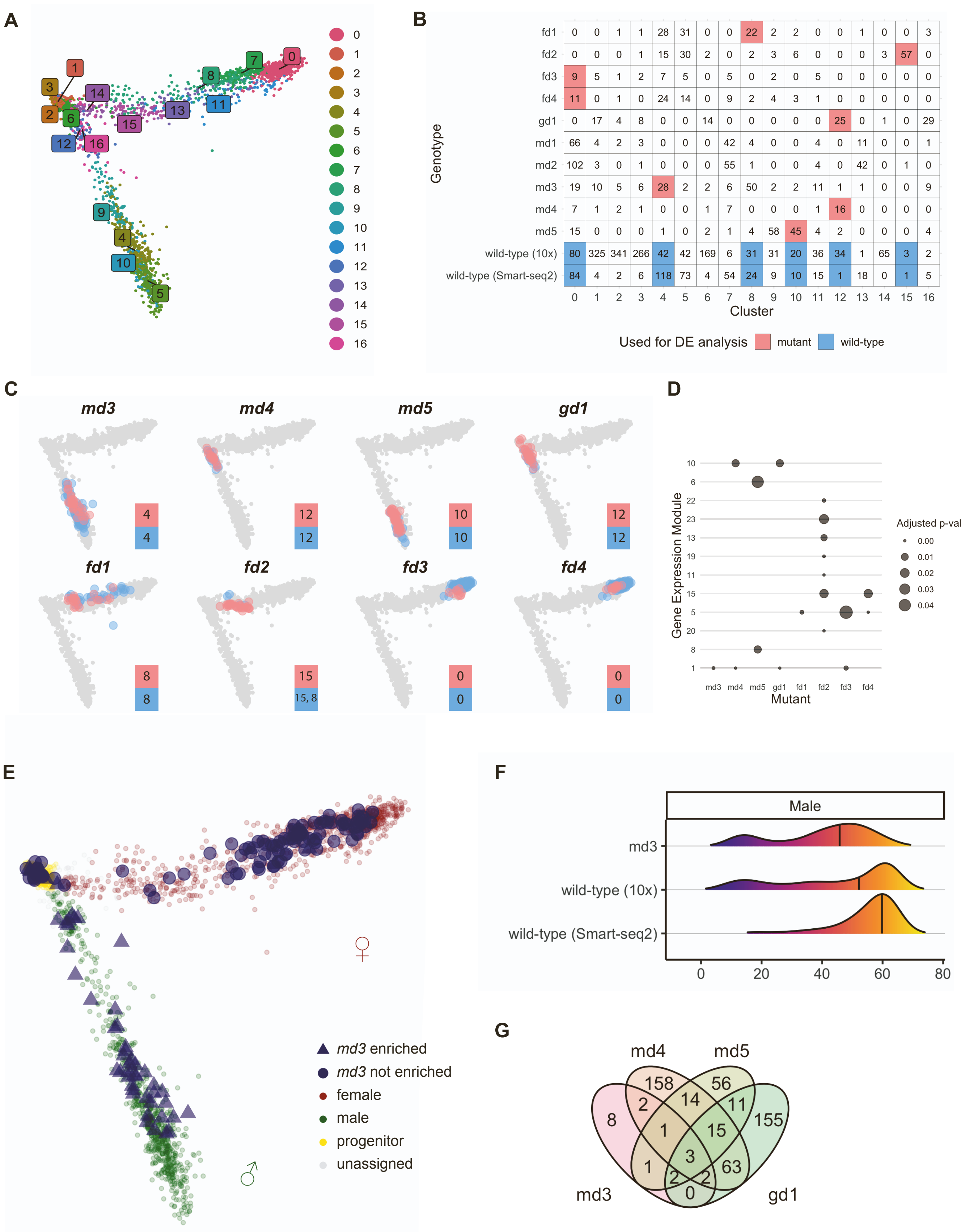

Figure S6. Differential gene expression testing. Related to Figure 4. (A) PCA plot showing the clusters that were generated after subsetting the branch cells in the wild type and mutant combined dataset. **(B)** Table showing the clusters that were used for differential expression analysis for each mutant (red) and the corresponding wild-type cells that were tested against (blue). The number of cells in each class is shown in the box. **(C)** PCA plots showing where the clusters that were used for differential expression analysis are located. The cluster number used is denoted in the boxes, top = mutant cell cluster, bottom = wild-type cell cluster. Using clusters that are similarly located on the PCA reduces the possibility that DE genes identified are due to differences in progression along the branch caused by the mutation rather than the mutation itself. **(D)** the

hypergeometric probability density function was used to identify enrichment of gene module genes within the differentially expressed genes for each mutant. points are sized by Benjamini-Hochberg Procedure adjusted p-values. **(E)** Disruption of *md3* was associated with a loss of male marker expression in the screen, and all 87 gametocytes subjected to Smart-seq2 analysis were females, indicative of a shift in sex ratio. However, in contrast to *md1* and *md2* mutants, male fertility was not completely lost in a genetic cross with a *hap2* mutant (Fig. 2F), and an *md3* KO clone was able to transmit the disrupted *md3* locus to mosquitoes and back to mice (Fig. 2E), suggesting there was a strongly reduced number of males in this mutant, which remained fertile. We therefore enriched rare cells expressing the male reporter gene by flow cytometry and subjected these to Smart-seq2 analysis. Smart-seq2 transcriptomes of enriched *md3* male gametocytes mapped onto the PCA plot from Fig. 4A. **(F)** Frequency distribution of transcriptomes over pseudotime for male md3 gametocytes enriched by flow sorting and wild type for each technology. **(G)** Numbers of genes with differences in transcript abundance in male gametocytes, compared to wild type as in Fig. 4C, but including the combined transcriptomes of male *md3* gametocytes enriched by flow sorting.

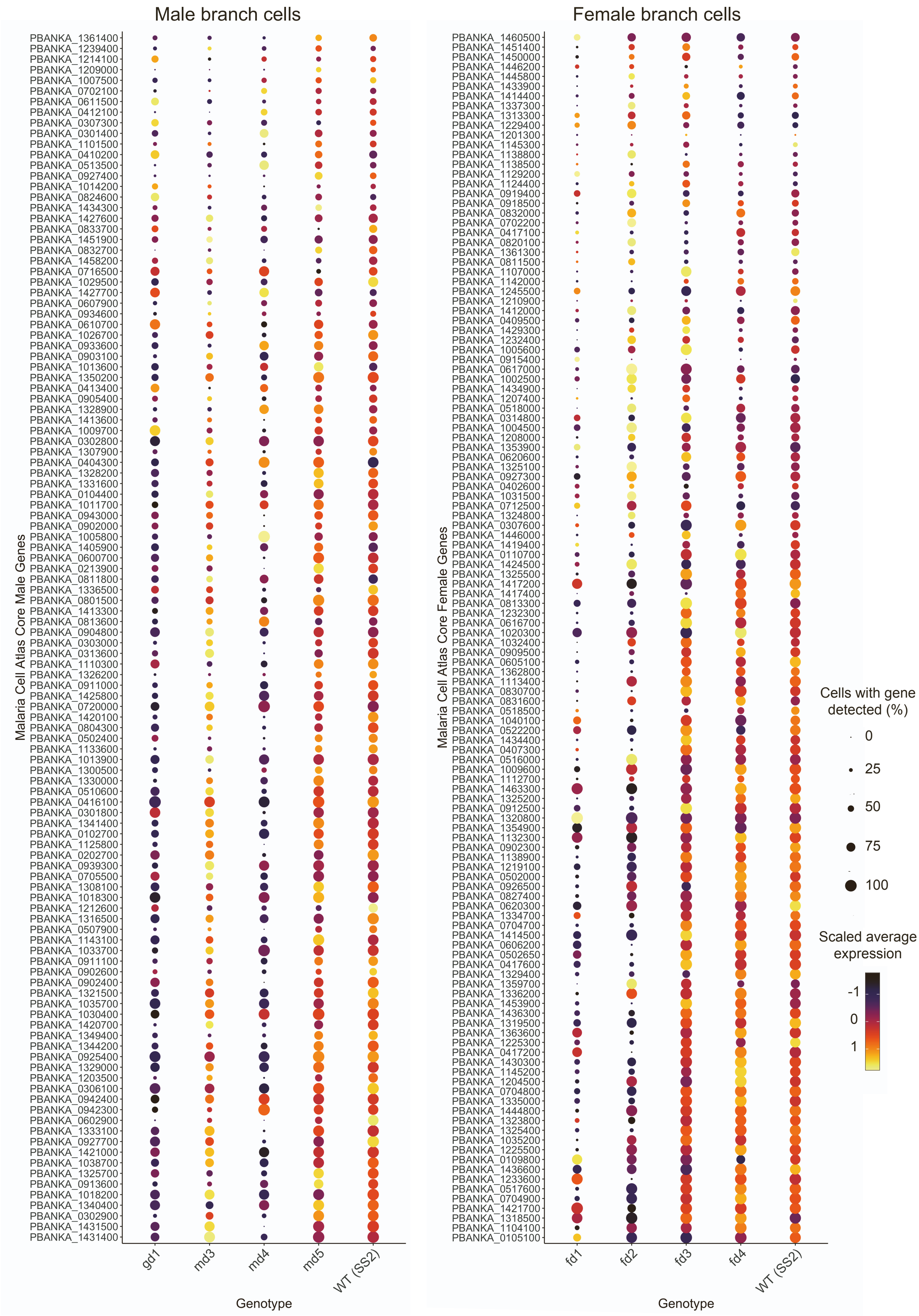

Figure S7. Core Sexual Genes Analysis. Related to Figure 4. The expression of core sexual genes defined in [S2] were plotted in each genotype within each sexual branch. WT (SS2) = wild-type (Smart-seq2).

Supplementary Tables

P. berghei gene ID	Name	*Pb* ap2-g early response gene	*Pf* 3D7 gene ID	Direct AP2G target (Josling et al. 2020)			van Biljon et al. (2019)
				stage-I gametocytes	sexual rings	schizonts	Cluster*
PBANKA_1302700	*md1*	YES	PF3D7_1438800	no	no	no	9
PBANKA_1447900	*md2*	YES	PF3D7_1233200	no	no	no	8
PBANKA_0413400	*md3*	YES	PF3D7_0315600	YES	no	no	7
PBANKA_0102400	*md4*	YES	PF3D7_0603600	YES	no	no	8
PBANKA_0716500	*md5*	no	PF3D7_0414500	no	no	no	8
PBANKA_0828000	*gd1*	YES	PF3D7_0927200	YES	no	no	8
PBANKA_1454800	*fd1*	YES	PF3D7_1241400	YES	no	no	8
PBANKA_0902300	*fd2*	YES	PF3D7_1146800	YES	no	no	8
PBANKA_1418100	*fd3*	YES	PF3D7_1319600	no	no	no	8
PBANKA_1435200	*fd4*	YES	PF3D7_1220000	no	no	no	9

Table S7. *Plasmodium falciparum* orthogs of investigated genes and some of their properties. Related to Figure 7. *P. berghei* early response gene in this study (responding to reprogramming within 1-2 hours). Direct AP2-G target: Has associated consensus binding peak in chromatin immunoprecipitation experiments in *P. falciparum* cultures whose differentiation was induced by AP2-G stabilisation [S4]. Cluster: Co-expression cluster during *P. falciparum* sexual differentiation according to the bulk microarray time course data in [S5]. Cluster descriptions are: 6-10 = increased during gametocytogenesis; 6-7 = transcripts maintained at increased levels throughout commitment and development; 7 = contains many differentiation genes; 8-10 with specific peaks during gametocytogenesis; 8 = transcripts involved in early stage development increasing from stage I-II; 9 = increased from intermediate stage III - IV; 10 = stage V transcripts.

Supplementary References

S1. Kent, R.S., Modrzynska, K.K., Cameron, R., Philip, N., Billker, O., and Waters, A.P. (2018). Inducible developmental reprogramming redefines commitment to sexual development in the malaria parasite Plasmodium berghei. Nat Microbiol 3, 1206–1213.

S2. Howick, V.M., Russell, A.J.C., Andrews, T., Heaton, H., Reid, A.J., Natarajan, K., Butungi, H., Metcalf, T., Verzier, L.H., Rayner, J.C., et al. (2019). The Malaria Cell Atlas: Single parasite transcriptomes across the complete life cycle. Science 365. https://doi.org/10.1126/science.aaw2619.

S3. Finak, G., McDavid, A., Yajima, M., Deng, J., Gersuk, V., Shalek, A.K., Slichter, C.K., Miller, H.W., Juliana McElrath, M., Prlic, M., et al. (2015). MAST: a flexible statistical framework for assessing transcriptional changes and characterizing heterogeneity in single-cell RNA sequencing data. Genome Biology 16. https://doi.org/10.1186/s13059-015-0844-5.

S4. Josling, G.A., Russell, T.J., Venezia, J., Orchard, L., van Biljon, R., Painter, H.J., and Llinás, M. (2020). Dissecting the role of PfAP2-G in malaria gametocytogenesis. Nature Communications 11. https://doi.org/10.1038/s41467-020-15026-0.

S5. van Biljon, R., van Wyk, R., Painter, H.J., Orchard, L., Reader, J., Niemand, J., Llinás, M., and Birkholtz, L.-M. (2019). Hierarchical transcriptional control regulates Plasmodium falciparum sexual differentiation. BMC Genomics 20, 920.
